# NOTCH3 signaling is essential for NF-κB activation in TLR-activated macrophages

**DOI:** 10.1038/s41598-020-71810-4

**Published:** 2020-09-09

**Authors:** Susana López-López, Eva María Monsalve, María José Romero de Ávila, Julia González-Gómez, Natalia Hernández de León, Francisco Ruiz-Marcos, Victoriano Baladrón, María Luisa Nueda, María Jesús García-León, Isabella Screpanti, María Pía Felli, Jorge Laborda, José Javier García-Ramírez, María José M. Díaz-Guerra

**Affiliations:** 1 Universidad de Castilla-La Mancha, Medical School/CRIB, Laboratory of Biochemistry and Molecular Biology, Department of Inorganic and Organic Chemistry and Biochemistry, UCLM, C/Almansa 14, 02008 Albacete, Spain; 2 Unidad Asociada de Biomedicina UCLM, Unidad Asociada CSIC, Albacete, Spain; 3 Universidad de Castilla-La Mancha, CRIB/Biomedicine Unit, Pharmacy School, UCLM/CSIC, C/Almansa 14, 02008 Albacete, Spain; 4 University Hospital Complex of Albacete, 02008 Albacete, Spain; 5 Department of Cell Biology and Immunology, Centro de Biología Molecular Severo Ochoa (CSIC), Universidad Autónoma de Madrid, 28049 Madrid, Spain; 6 Department of Molecular Medicine, Sapienza University, Viale Regina Elena 291, 00161 Roma, Italy; 7 Department of Experimental Medicine, Sapienza University, Viale Regina Elena 324, 00161 Rome, Italy; 8 Present Address: INSERM UMR_S1109, Tumor Biomechanics, 67000 Strasbourg, France; 9 Present Address: Université de Strasbourg, 67000 Strasbourg, France; 10 Present Address: Fédération de Médecine Translationnelle de Strasbourg (FMTS), 67000 Strasbourg, France

**Keywords:** Inflammation, Innate immune cells, Innate immunity

## Abstract

Macrophage activation by Toll receptors is an essential event in the development of the response against pathogens. NOTCH signaling pathway is involved in the control of macrophage activation and the inflammatory processes. In this work, we have characterized NOTCH signaling in macrophages activated by Toll-like receptor (TLR) triggering and determined that DLL1 and DLL4 are the main ligands responsible for NOTCH signaling. We have identified ADAM10 as the main protease implicated in NOTCH processing and activation. We have also observed that furin, which processes NOTCH receptors, is induced by TLR signaling in a NOTCH-dependent manner. NOTCH3 is the only NOTCH receptor expressed in resting macrophages. Its expression increased rapidly in the first hours after TLR4 activation, followed by a gradual decrease, which was coincident with an elevation of the expression of the other NOTCH receptors. All NOTCH1, 2 and 3 contribute to the increased NOTCH signaling detected in activated macrophages. We also observed a crosstalk between NOTCH3 and NOTCH1 during macrophage activation. Finally, our results highlight the relevance of NOTCH3 in the activation of NF-κB, increasing p65 phosphorylation by p38 MAP kinase. Our data identify, for the first time, NOTCH3 as a relevant player in the control of inflammation.

## Introduction

Macrophages are crucial cells for the innate immune system; they are present in a large variety of locations, and play distinct functions as determined by their developmental origins and by the nature of the activators and the microenvironment. Macrophages’ functions range from pro-inflammatory to anti-inflammatory-pro-resolution-deactivation; these activities play a relevant role in the onset of inflammation and coexist in the course of the immune response^1^. Increasing evidence indicates the involvement of NOTCH receptor signaling in the regulation of the innate immune response, especially in the control of macrophage activation and polarization. Different studies have reported that the expression of several cytokines, such as IL-6, IL-12 and IL-10, in response to macrophage activation by Toll-like receptors (TLRs), depends on the activity of NOTCH receptors^2–4^. Moreover, in different models of inflammatory disease, increased expression of NOTCH receptors and increased NOTCH signaling have been reported to cause pathological inflammation^5^.

NOTCH signaling is a cell–cell communication signaling pathway regulating cell fate decisions in developing embryos and adult animal tissues. Four NOTCH receptors (NOTCH1-4) have been described in mammals. NOTCH receptors are single-pass, type I transmembrane heterodimers, generated as a single chain precursor that is cleaved by furin in the trans-Golgi network. Furin is a calcium-dependent serine protease that, in addition to cleave NOTCH receptors^6^, activates several proteases involved in NOTCH signaling, including ADAM10 and ADAM17^7,8^. The expression levels of furin differ in different cell types, and also depend on the degree of cell differentiation and activation^9^.

The NOTCH pathway is initiated by the binding of transmembrane ligands from the Delta/Serrate/LAG-2 (DSL) family, DLL and JAGGED to NOTCH receptors. In mammals, three Delta-like ligands (DLL1, DLL3 and DLL4) and two JAGGED ligands (JAG-1 and JAG-2) have been identified. The canonical NOTCH activation pathway depends on a proteolytic cascade induced by ligand binding that triggers a first cleavage just outside the membrane mediated by ADAM metalloproteases, followed by a second cleavage, just inside the inner leaflet of the cell membrane, by a presenilin-γ-secretase complex. These proteolytic cleavages release the intracellular domain of the NOTCH receptor (NICD), which then translocates into the nucleus and promotes the displacement of a repressor complex, favoring the activation of its target genes^10^.

The four mammalian NOTCH proteins display both overlapping and distinct tissue distributions, as well as both redundant and distinct functions^11^. *Notch3* and *Notch4* present a more restricted tissue distribution than *Notch1* and *Notch2*^11,12^, which possibly explains why targeted deletions of murine *Notch1* or *Notch2* lead to embryonic lethality^12,13^, contrary to deletions of *Notch3* or *Notch4*^14,15^. Some studies indicate that different intracellular NOTCH proteins read binding site orientations and distributions on the target promoters differently^16^.

The individual contribution of each receptor to macrophage activation remains unclear, although most studies point to NOTCH1 as the main receptor implicated in macrophage pro-inflammatory activation, mainly because it is highly expressed after activation of macrophages by Toll receptors^2^. Furthermore, the relevance of the different niche-derived NOTCH ligands in macrophage activation has not been clearly evaluated. Indeed, increasing evidence points to ligand-independent NOTCH signaling in some NOTCH-mediated processes^17^.

Using functional studies in vivo and in cultured mammalian cells, different groups have established that the metalloprotease ADAM10, but not ADAM17, is essential for ligand-induced extracellular cleavage of NOTCH1 and 2^18,19^. On the other hand, ADAM17 has been related to ligand-independent NOTCH signaling^20^.

In this study, we have characterized NOTCH signaling in pro-inflammatory macrophages activated by TLR, evaluating the relevance of the different NOTCH ligands in the niche-macrophage interaction, characterizing the ADAM proteases implicated in NOTCH activation, and assessing the role of each NOTCH receptor in this process. Our results identify for the first time NOTCH3 as a relevant player in the control of inflammation, highlighting the importance of NOTCH3 for the activation of NF-κB and in pro-inflammatory macrophage development.

## Results

### DLL4 and DLL1, but not JAGGED1, increase NOTCH signaling in activated macrophages

**Figure 1:**
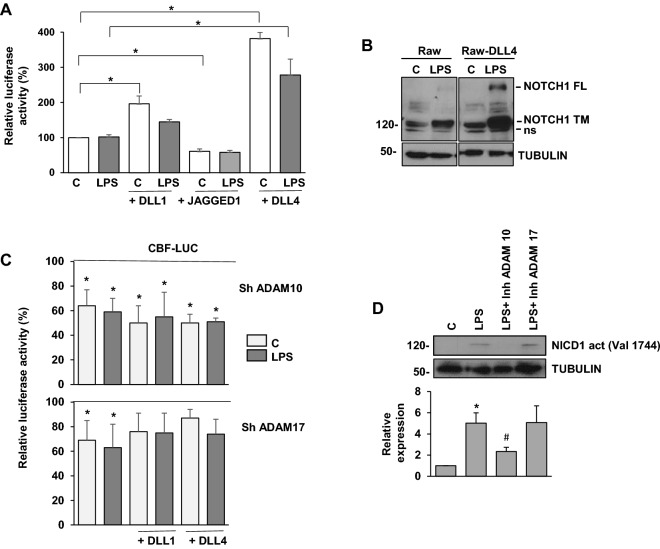
Analysis of the ligands and proteases responsible for NOTCH signaling after macrophage activation by TLR-4. (**A**) NOTCH transcriptional activity analysis in Raw 264.7 cells transiently transfected with a CBF luciferase reporter gene (CBF-luc) and cultured over OP9 cells expressing human JAGGED1, DLL1 or DLL4. One day after transfection, cells were stimulated with LPS (100 ng/ml) for 24 h before analysis. The means ± SD of three independent experiments are shown. One-way ANOVA/Bonferroni’s post-tests were performed. *p < 0.05, with respect to their corresponding controls. (**B**) Western blot analysis of NOTCH1 expression in control and DLL4 overexpressing Raw cells activated with LPS. A representative experiment is shown. FL: full length; TM: transmembrane. ns: non-specific. (**C**) NOTCH-dependent transcriptional activity analysis in Raw264.7 cells transiently transfected with a CBF luciferase reporter (CBF-luc) and cultured over control or OP9 cells expressing human DLL1 and DLL4 in the presence of control, ADAM10 or ADAM17 sh RNAs, as indicated. One day after transfection, cells were stimulated with LPS (100 ng/ml) for 24 h before analysis. The means ± SD of three independent experiments are shown. One-way ANOVA/Bonferroni’s post-tests were performed. *p < 0.05, with respect to their corresponding controls. (**D**) Evaluation of the active NOTCH1 intracellular domain (NICD1-valine 1744) expression by Western blot in Raw264.7 cells treated or not with the ADAM10 or ADAM17 inhibitors GI254023X (5 μM) and GW280264X (5 μM), respectively, and activated with LPS for 24 h. A representative experiment is shown. Quantitation of three different experiments is shown on the lower panel. Means ± SD of three independent experiments are shown. One-way ANOVA/Bonferroni’s post-tests were performed. *p < 0.05, with respect to control cells, ^#^*p* < 0.05, with respect to cells activated with LPS.

We first characterized NOTCH receptor signaling after macrophage activation by LPS-dependent Toll receptor triggering and evaluated the relevance of the different NOTCH ligands in the niche-macrophage interaction. To do that, OP9 stromal cells expressing the human NOTCH ligands DLL1, DLL4 or JAGGED were co-cultured with control or activated Raw 264.7 macrophages to analyze their effects on the activation of a NOTCH-dependent luciferase reporter gene. As shown in Fig. 1A, expression of DLL4 and, to a lesser extent, DLL1 in OP9 cells, increased basal and LPS-induced NOTCH signaling. Conversely, JAGGED1 appeared to be unable to activate NOTCH signaling, and it rather seemed to induce a reduction of Notch activity. Accordingly, higher levels of NOTCH1 were observed when DLL4-expressing Raw 264.7 cells were treated with LPS (Fig. 1B), since NOTCH1 expression depends on NOTCH signaling^21^.

### ADAM10 is the main protease responsible for NOTCH signaling after TLR4 macrophage activation

Different proteases from the ADAM family, mainly ADAM10 and ADAM17, have been reported to cleave NOTCH receptors and allow the subsequent liberation of NICD by γ-secretase. We evaluated the relevance of these two proteases in NOTCH signaling in macrophages activated through TLR4 triggering. To do that, we measured the activity of a CBF1-dependent luciferase reporter gene after DLL1- or DLL4-mediated NOTCH activation, using specific enzymatic inhibitors and shRNAs of ADAM10 and ADAM17^21^. As Fig. 1C shows, the presence of an ADAM10 shRNA clearly diminished CBF1-dependent reporter activity, both in basal and LPS-activated conditions, for either DLL1 or DLL4-induced cells. However, when using ADAM17 shRNA, the only significant effect was observed in basal and LPS conditions, and no differences were detected with either of the ligands. To confirm these results, we used specific ADAM10 (GI254023X) or ADAM17 (GW280264X) inhibitors and measured the release of the active NOTCH1 intracellular domain (NICD1), in order to analyze their effect on NOTCH1 activation levels. The ADAM10 inhibitor completely blocked the release of NICD1, while the ADAM17 inhibitor had no effect (Fig. 1D). Taken together, these results show that ADAM10 is the main protease implicated in NOTCH signaling after TLR activation.

### Furin expression is upregulated by TLR signaling in a NOTCH-dependent manner

**Figure 2:**
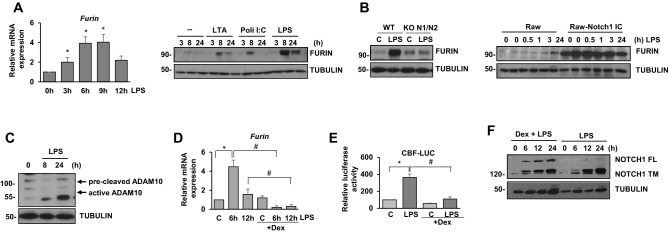
Analysis of furin expression after macrophage activation by TLR-4 signaling. (**A**) qRT-PCR (left panel) and Western blot (right panel) analyses of furin expression in Raw264.7 cells induced with LTA, poli I:C or LPS, for the indicated times. Means ± SD of three independent experiments are shown for qRT-PCR. One-way ANOVA/Bonferroni’s post-tests were performed. Statistical significance was determined at the level of *p* < 0.05; for Western blot, a representative experiment of three is shown. (**B**) Analysis of furin expression by Western blot in control and *Notch1/2*^−/−^ macrophages activated by LPS for 12 h (left panel), or in Raw cell transfected with the intracellular domain of NOTCH1 (Raw-NICD1) or the vector (Raw) (right panel). A representative experiment of three is shown in each case. (**C**) Analysis of ADAM10 expression and activation by Western blot in macrophages activated by LPS for different times. A representative experiment is shown. (**D**) qRT-PCR analysis of furin expression in LPS activated macrophages in the presence or in the absence of 1 µM dexamethasone (Dex). The means ± SD of three independent experiments are shown. One-way ANOVA/Bonferroni’s post-tests were performed. *p < 0.05, compared to cells not treated with LPS. ^#^*p* < 0.05, compared to cells not treated with dexamethasone. (**E**) NOTCH transcriptional activity analysis in Raw264.7 cells transiently transfected with a CBF luciferase reporter gene (CBF-luc) and stimulated with LPS (100 ng/ml) and/or dexamethasone (1 µM) for 24 h before analysis. The means ± SD of three independent experiments are shown. One-way ANOVA/Bonferroni’s post-tests were performed. *p < 0.05, compared to cells not treated with LPS. #*p* < 0.05, compared to cells not treated with dexamethasone. (**F**) Analysis of NOTCH1 expression in macrophages activated with LPS in the presence or in the absence of 1 µM dexamethasone for different times. A representative experiment out of three performed independently is shown. FL: full length; TM: transmembrane.

NOTCH receptors are first cleaved by furin in the Golgi apparatus to produce the biologically active heterodimers. Furin also cleaves and activates ADAM10 and ADAM17^10^. Previous works have shown that furin expression in melanoma cells depends on NOTCH signaling^22^, and that TLR signaling increases furin levels^23^. To characterize the role of furin in macrophages, we first analyzed whether its expression was activated by LPS. As shown in Fig. 2A, increased furin mRNA levels were observed after TLR-4 activation, reaching maximal expression after 6–9 h. We next analyzed if macrophage activation with different TLR agonists changed furin expression. Our data showed that macrophage activation by TLR-2, 3 or 4 receptors induced furin expression. Finally, we evaluated the contribution of NOTCH signaling to the expression of furin induced by TLRs, by using WT and Notch1/Notch2 KO macrophages activated with LPS. As shown in the left panel of Fig. 2B, our results clearly indicate that induction of furin by TLR-4 activation depends on NOTCH signaling; moreover, the expression of the intracellular domain of Notch1 in Raw cells increased drastically the expression of furin, as shown in the right panel of Fig. 2B, confirming the role of Notch signaling in furin expression.

Increased furin expression has been related with higher NOTCH processing and signaling^6^. We investigated whether the induction of furin expression in macrophages by TLR signaling could enhance ADAM10 processing and activation. As Fig. 2C shows, increased ADAM10 processing was clearly observed after LPS treatment. We also observed that induction of furin mRNA expression by TLR signaling was inhibited by the anti-inflammatory drug dexamethasone (Fig. 2D) and, in agreement with this effect, dexamethasone also inhibited NOTCH signaling (Fig. 2E) and the processing of the full length NOTCH1 receptor (Fig. 2F).

Therefore, with all these results we might speculate that the induction of furin expression by TLR signaling in macrophages could enhance ADAM10 processing and activation, thus favoring ligand-dependent NOTCH signaling. Moreover, NOTCH signaling enhances LPS-dependent furin expression, which could lead to a positive activation loop in this signaling pathway. Glucocorticoids, such as dexamethasone, could inhibit this process by blocking the expression of furin.

### Contribution of each NOTCH receptor to NOTCH signaling in TLR-activated macrophages

**Figure 3:**
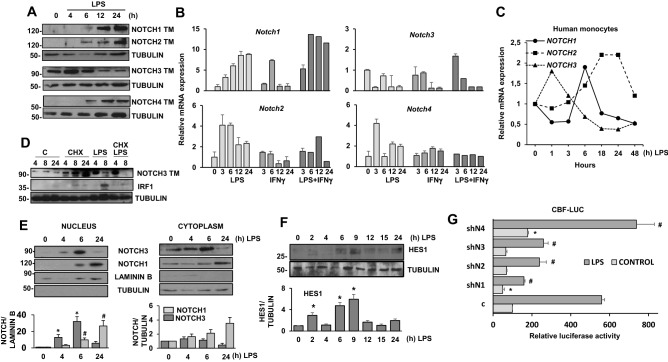
Analysis of NOTCH receptor expression and signaling after macrophage activation by TLR-4 agonists. (**A**) Western blot analysis for the expression of NOTCH receptors in peritoneal macrophages activated with LPS for different times. β-tubulin expression was used as a loading reference. The image is representative of five independent experiments. (**B**) qRT-PCR analysis of *Notch* gene expression in peritoneal murine macrophages activated with LPS (100 ng/ml) in the presence or in the absence of IFNγ (20 U/ml) for different times. Means ± SD of three independent experiments are shown. (**C**) qRT-PCR analysis of *Notch1*, *2* and *3* mRNA expression in human monocytes activated with LPS (100 ng/ml) for different times. A representative experiment of five is shown. (**D**) Western blot analysis of NOTCH3 expression in macrophages activated with LPS for different times after cycloheximide (2.5 µg/ml) treatment for 12 h. Expression of IRF1 was used as a control and β-tubulin expression was used as a loading reference. Image is representative of three independent experiments. (**E**) Analysis of NOTCH1 and NOTCH3 translocation to the nucleus in macrophages activated with LPS (100 ng/ml) for different times. Nuclear extracts are shown on the left panel, using laminin as a loading control. Cytoplasmic extracts are shown on the right panel, using tubulin as a loading control. Quantitation of three different experiments is shown. One-way ANOVA/Bonferroni’s post-tests were performed. *p < 0.05 (NOCTH3) and #*p* < 0.05 (NOTCH1) translocation compared to non LPS-treated cells. (**F**) Analysis by Western blot of HES1 expression in peritoneal macrophages activated with LPS for different times. β-tubulin expression was used as a loading reference. Quantitation of three different experiments is shown on the lower panel. Means ± SD of three independent experiments are shown. One-way ANOVA/Bonferroni’s post-tests were performed. Statistical significance was determined at the level of *p* < 0.05. (**G**) NOTCH transcriptional activity analysis in Raw 264.7 cells transiently co-transfected with a CBF luciferase reporter (CBF-luc), and shRNAs specific for the different *Notch* receptor genes. One day after transfection, cells were stimulated with LPS for 24 h before analysis. The means ± SD of six independent experiments are shown. One-way ANOVA/Bonferroni’s post-tests were performed. *p < 0.05 compared to control, untreated cells. #*p* < 0.05 compared to control, LPS-treated cells.

Previous works from our group and others have shown the implication of NOTCH signaling in the function of pro-inflammatory activated macrophages^2,24–26^. In fact, focus has been directed to NOTCH1, as it was thought to be the most relevant NOTCH receptor in this process^2^. To dissect the contribution of the four NOTCH receptors, we analyzed by Western Blot and qPCR the expression of all *Notch* receptor genes in control and LPS-activated macrophages in the presence or in the absence of IFNγ. Western blot analysis of resting macrophages showed that the only receptor with a basal expression was NOTCH3, whereas NOTCH1, NOTCH2 and NOTCH4 expression was not detected. Moreover, macrophage activation through TLR-4 greatly induced the expression of NOTCH1, NOTCH2 and NOTCH4 receptors, as can be observed by the increase of their transmembrane domain levels (Fig. 3A) and as previously described^2,3^. Interestingly, NOTCH3 transmembrane domain levels increased at early times after activation by TLR-4 receptor; however, after longer activation times (12 h), a decrease in NOTCH3 levels was observed, parallel to the induction of NOTCH1, NOTCH2 and NOTCH4 expression (Fig. 3A).

The analysis of mRNA expression by qRT-PCR showed that, while the expression levels of *Notch1* and *Notch2* mRNA increased strongly after TLR activation, the level of expression of *Notch3* and *Notch4* mRNA only increased slightly (Fig. 3B). Interestingly, IFNγ mainly affected *Notch1* expression, enhancing the activating effect of LPS (Fig. 3B). In human monocytes, the mRNA expression of *NOTCH1*, 2 and *3* showed a pattern similar to that of murine macrophages, with higher expression of *NOTCH3* at early times after LPS activation*,* followed by a drop in its expression, and peaks of *NOTCH1* and *NOTCH2* expression at later times (Fig. 3C). Thus, NOTCH3 seems to be the main NOTCH receptor present in differentiated, resting macrophages, whereas the activation by TLR receptors increases the expression of the other three NOTCH receptors.

The rapid surge in NOTCH3 protein levels observed in the first hours after LPS activation was produced without any detectable increase in mRNA expression levels. To analyze that apparent discrepancy, we quantified NOTCH3 expression levels in the presence of the protein synthesis inhibitor cycloheximide (CHX). As Fig. 3D shows, increased NOTCH3 levels were detected in the presence of CHX, arguing in favor of a continuous modulation of NOTCH3 protein levels by post-translational processing and/or stabilization. As expected, cycloheximide treatment led to a clear inhibition of IRF1 expression. The fact that after 4 h of LPS treatment, the induction of NOTCH3 expression was not affected by the presence of CHX, suggests that the LPS-induced increase in NOTCH3 expression depends more on the stabilization of the protein than on its new synthesis. However, after 8 h of treatment, a fall in NOTCH3 expression was observed. This decrease was significantly greater in macrophages treated with LPS plus CHX than in those treated with LPS alone; thus, the blockade of new protein synthesis accelerated the fall in NOTCH3 expression.

As Notch receptor signaling implies NICD release and translocation to the cell nucleus, we evaluated the translocation to the nucleus of the intracellular domains of NOTCH1 and NOTCH3 after LPS treatment (Fig. 3E). In order to detect any possible cross-contamination of the protein extracts, the cytoplasmic fractions, with their corresponding tubulin loading controls, were analyzed and are shown on the right panel. As shown on the left panel of Fig. 3E, NOTCH3 accumulation in the nucleus of macrophages preceded that of NOTCH1.

We also evaluated the expression of the NOTCH target protein HES1 in LPS-activated macrophages by Western blot. As shown in Fig. 3F, a slight increase in HES1 levels was observed 2 h after treating with LPS, before any NOTCH1, NOTCH2 and NOTCH4 expression was detected, arguing for a role of NOTCH3 in its early expression. However, the highest expression of HES1 was detected at 6–9 h after LPS treatment, when all NOTCH receptors were expressed in the activated macrophages. That made us wonder what the contribution of each NOTCH receptor to NOTCH signaling was in activated macrophages. To discern that, we used specific *Notch1-4* shRNAs, aimed to diminish the expression of each NOTCH receptor. We transfected Raw 264.7 cells with the corresponding most effective *Notch1-4* shRNAs and a CBF-Luc reporter plasmid, and measured luciferase activity. As shown in Fig. 3G, shRNAs specific to *Notch1*, *Notch2* or *Notch3* significantly reduced LPS-induced CBF reporter activity, whereas in untreated cells, the only significant reduction was caused by the shRNA specific to NOCTH1. Interestingly, *Notch4* silencing seemed to increase NOTCH activity in untreated and LPS-treated cells. Among the four receptors, NOTCH1 seemed to be the most effective in diminishing CBF reporter activity. Taken together, our results suggest that NOTCH3 is the most relevant NOTCH receptor soon after TLR activation, but other NOTCH receptors, mostly NOTCH1 and NOTCH2, appear to be more relevant to control the expression of NOTCH target genes at later times.

### NOTCH1 modulates NOTCH3 expression through ZEB1 in TLR-activated macrophages

**Figure 4:**
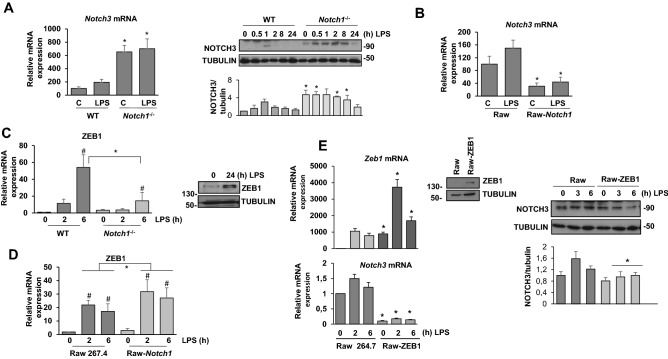
Analysis of Notch3 expression and signaling in macrophages activated by TLR-4. (**A**) qRT-PCR (left panel) and Western blot (right panel) analysis of *Notch3* expression in WT and *Notch1* KO peritoneal murine macrophages activated with LPS (100 ng/ml) for different times. Means ± SD of at least three independent experiments are shown. One-way ANOVA/Bonferroni’s post-tests were performed. *p < 0.05 in any treatment condition compared to WT macrophages. (**B**) qRT-PCR analysis of *Notch3* expression in Raw 264.7 cells transiently transfected with a *Notch1* expression vector (Raw-Notch1) or with the corresponding empty vector (Raw). One day after transfection, cells were stimulated with LPS for 24 h before analysis. The means ± SD of three independent experiments are shown. One-way ANOVA/Bonferroni’s post-tests were performed. *p < 0.05 compared to the empty vector-transfected cells. (**C**) (Left panel) qRT-PCR analysis of *Zeb1* mRNA expression in WT and *Notch1* KO peritoneal murine macrophages activated with LPS (100 ng/ml). Means ± SD of at least three independent experiments are shown. One-way ANOVA/Bonferroni’s post-tests were performed. #*p* < 0.05 compared to control conditions, *p < 0.05 compared to WT macrophages. (Right panel) Western blot analysis of ZEB1 expression in WT macrophages activated with LPS for 24 h. A representative experiment of two is shown. (**D**) qRT-PCR analysis of *Zeb1* expression in Raw 264.7 cells transiently transfected with a *Notch1* expression vector (Raw-*Notch1*) or the corresponding empty vector (Raw). One day after transfection, cells were stimulated with LPS for 24 h before analysis. The means ± SD of four independent experiments are shown. One-way ANOVA/Bonferroni’s post-tests were performed. #*p* < 0.05 compared to control conditions, *p < 0.05 compared to the empty vector-transfected cells. (**E**) (Left panels) qRT-PCR analysis of *Zeb1* (upper left panel) and *Notch3* (lower left panel) expression, (middle panel) Western blot analysis of ZEB1 expression in Raw cells transfected with control (Raw) or ZEB1 expression vector (Raw-ZEB1), and (right panels) Western blot analysis of NOTCH3 expression in Raw cells transiently transfected with a *Zeb1* expression vector (Raw-ZEB1) or the corresponding empty vector (Raw). One day after transfection, cells were stimulated with LPS for 24 h before analysis. The means ± SD of three independent experiments are shown. One-way ANOVA/Bonferroni’s post-tests were performed. *p < 0.05 compared to the empty vector-transfected cells.

The analysis of the expression of NOTCH receptors showed that NOTCH3 is present in resting, differentiated macrophages, and that its protein levels decreased as the protein levels of all the other NOTCH receptors are induced due to TLR activation (Fig. 3A). An interaction between NOTCH1 and NOTCH3 has been implicated in multiple differentiation process. Indeed, in squamous cell carcinoma, NOTCH1 induces the expression of Zinc finger E-box-binding homeobox 1 (ZEB1), a transcription factor that acts as a negative regulator of *Notch3* gene expression^27^. We observed that *Notch3* expression was increased in macrophages lacking NOTCH1 both at the mRNA and protein levels (Fig. 4A). On the contrary, Raw 264.7 cells overexpressing *Notch1* showed lower levels of *Notch3* mRNA (Fig. 4B). We wondered whether ZEB1 could be responsible for this effect, as *Zeb1* gene expression can be induced in an NF-κB-dependent manner in tumoral cells activated by mucin-1^28^. When we analyzed LPS-activated macrophages, we observed that ZEB1 protein levels increased (Fig. 4C left panel), accordingly with an elevation of its corresponding mRNA (Fig. 4C left panel). This mRNA induction was lower in *Notch1*^−/−^ cells, whereas it increased when *Notch1* was overexpressed in Raw 264.7 cells (Fig. 4C right panel and D).

To evaluate whether the higher than normal *Notch3* expression levels of *Notch1*^−/−^ macrophages could be mediated by a lower than normal ZEB1 expression, we transfected *Zeb1* in Raw 264.7 cells (Raw-ZEB1 cells) and analyzed the effect of *Zeb1* overexpression on *Notch3* gene expression. Raw-ZEB1 cells expressed high levels of ZEB1 protein (Fig. 4E left panel), and when we analyzed the *Zeb1* mRNA levels by quantitative RT-PCR, we observed a higher expression level both in control and in LPS-treated Raw-ZEB1 cells (Fig. 4E). We then evaluated *Notch3* expression, by measuring mRNA and protein in control and Raw-ZEB1 cells activated by LPS. As Fig. 4E (right and lower panel) shows, when ZEB1 levels were higher, the induction of the expression of *Notch3* by LPS*,* both at mRNA and protein levels, was lower. Therefore, we conclude that NOTCH1 controls the expression of NOTCH3 in LPS-activated macrophages, and suggest that this effect could be mediated, at least in part, through the modulation of *Zeb1* expression, which, in turn, would inhibit *Notch3* expression.

### NOTCH3 favors the activation of NF-κB and promotes pro-inflammatory gene expression in TLR-4-activated macrophages

**Figure 5:**
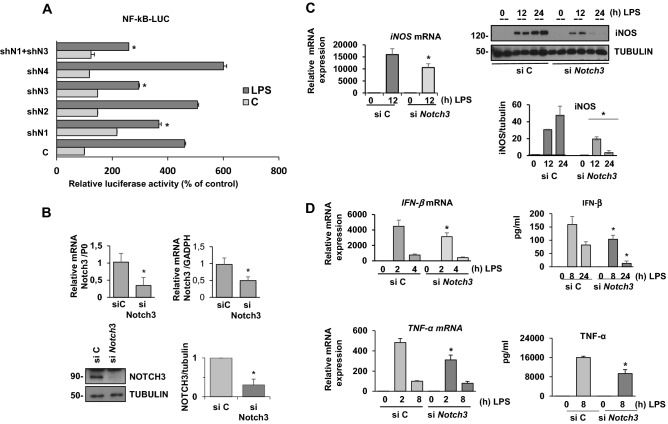
NOTCH3 favors the activation of NF-κB and promotes pro-inflammatory gene expression in TLR-4 activated macrophages. (**A**) Analysis of luciferase activity in Raw 264.7 cells transiently transfected with an NF-κB luciferase reporter (NF-κB-luc) and specific shRNAs for the different *Notch* receptor genes alone or in combination. One day after transfection, cells were stimulated with LPS (100 ng/ml) for 24 h before analysis. The means ± SD of three independent experiments are shown. One-way ANOVA/Bonferroni’s post-tests were performed. *p < 0.05 compared to the control shRNA-transfected cells. (**B**) Quantitative PCR (upper panel) and Western blot (lower panel) analysis of NOTCH3 expression in Raw 264.7 cells transfected with scrambled or *Notch3* siRNA for 48 h. RNA expression was referred to either P0 or GAPDH housekeeping controls. Protein levels were referred to tubulin loading control. Quantitation of three different experiments is shown. *p < 0.05 (T student analysis). (**C**) qRT-PCR (left panel) and Western blot (right panel) analysis of iNOS expression in macrophages transfected with scrambled (siC) or *Notch3* siRNA (siNotch3) for 48 h and activated with LPS for the indicated times. In Western blot, duplicates of different times of activation are shown. The quantitation of the Western blot is shown below. Means ± SD of at least three independent experiments are shown. One-way ANOVA/Bonferroni’s post-tests were performed, *p < 0.05 compared to the corresponding scrambled siRNA. (**D**) qRT-PCR (left panels) and ELISA analysis (right panels) of TNFα and IFNβ expression in macrophages transfected with scrambled (si C) or *Notch3* siRNA (siNotch3) for 48 h and activated with LPS for the indicated times. Means ± SD of at least three independent experiments are shown. One-way ANOVA/Bonferroni’s post-tests were performed. *p < 0.05, compared to the corresponding scrambled siRNA.

We wanted to study whether the different NOTCH receptors played similar roles in TLR-activated macrophages. NOTCH signaling modulates pro-inflammatory gene expression by different mechanisms (reviewed in^29^), including changes in NF-κB activity^25^. We evaluated the effect of each NOTCH receptor on NF-κB activity in Raw 264.7 cells transfected with specific *Notch1-4* shRNAs and with an NF-κB reporter gene, after LPS activation. As Fig. 5A shows, LPS activation increased NF-κB reporter activity in macrophages, but this LPS-induced NF-κB activity was inhibited when *Notch3*, and to a lesser extent *Notch1*, were silenced; however, neither *Notch2* nor *Notch4* silencing inhibited NF-κB activity. Previous results from our group and others^3,25^ revealed that NOTCH1 modulates NF-κB activity in LPS-activated macrophages. For that reason, we decided to evaluate the effect on NF-κB activity of silencing both NOTCH1 and NOTCH3 receptors in LPS-activated Raw 267.4 cells. As shown in the Fig. 5A, simultaneous silencing of both receptors produced a greater drop in NF-κB activity than the blockade of each individual receptor, suggesting that NOTCH1 and NOTCH3 individually contribute to modulate NF-κB, and that both receptors are necessary for maximal NF-κB activity after TLR-4 receptor activation.

To further analyze the prominent role of NOTCH3 on NF-κB activation, we diminished NOTCH3 expression by using specific siRNA (Fig. 5B) and evaluated the expression of pro-inflammatory NF-κB-dependent genes, such as *iNOS*, *TNF-α* and *IFNβ* in peritoneal macrophages activated by LPS. Specific *Notch3* siRNAs diminished the mRNA and protein expression of *iNOS*, *TNF-α* and *IFN-*β evaluated by quantitative RT-PCR, Western blot or ELISA (Fig. 5C,D).

**Figure 6:**
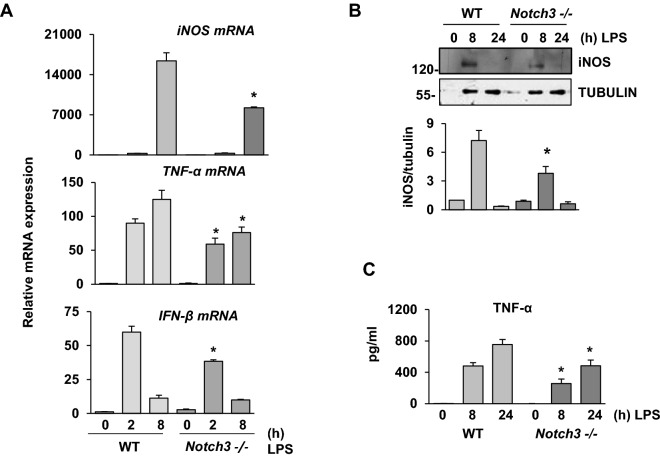
*Notch3*^*−/−*^ activated macrophages show lower expression of pro-inflammatory genes. qRT-PCR (**A**), Western blot (**B**) and ELISA analysis (**C**) of iNOS, TNF-α and IFN-β expression in WT or *Notch3*^*−/−*^ bone marrow-derived macrophages activated with LPS for the indicated times. Quantitation of the Western blot is shown. Means ± SD of at least three independent experiments are shown. One-way ANOVA/Bonferroni’s post-tests were performed. Statistical significance was determined at the level of *p* < 0.05.

To confirm these results, we compared the expression of the NF-κB dependent genes in WT and *Notch3*^*−/−*^ macrophages activated with LPS. As Fig. 6 shows, a lower expression of *iNO*S*, TNF-α* and *IFN-*β was observed in *Notch3*^*−/−*^ macrophages (Fig. 6A). In this regard, LPS-induced *Notch3*^*−/−*^ macrophages displayed a reduced protein level of *iNO*S (Fig. 6B). Additionally, TNFα release greatly decreased in *Notch3*^*−/−*^ macrophages after LPS activation (Fig. 6C). All these results argue for a role of NOTCH3 in the pro-inflammatory activation of macrophages by TLR-4.

### NOTCH3 modulates NF-κB phosphorylation by increasing p38 activation

**Figure 7:**
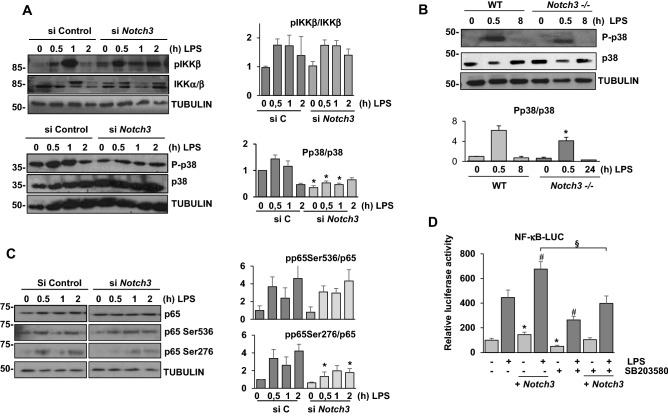
NOTCH3 modulates the phosphorylation and activation of p38 MAP kinase, thus controlling p65 phosphorylation on Ser276 after TLR signaling. (**A**) Western blot analysis of IKKα/β and p38 phosphorylation and expression in peritoneal macrophages transfected with scrambled (siC) or *Notch3* siRNA for 48 h and activated with LPS for the indicated times. Quantitation of Western blots is shown. Means ± SD of at least three independent experiments are shown. One-way ANOVA/Bonferroni’s post-tests were performed. Statistical significance was determined at the level of *p* < 0.05. (**B**) Western blot analysis of p38 phosphorylation and expression in WT and *Notch3*^*−/−*^ in bone marrow-derived macrophages activated with LPS for the indicated times. Quantitation of Western blot is included. Means ± SD of at least three independent experiments are shown. One-way ANOVA/Bonferroni’s post-tests were performed. Statistical significance was determined at the level of *p* < 0.05. (**C**) Western blot analysis of p65 phosphorylation on Ser536 and Ser276 in peritoneal macrophages transfected with scrambled (siC) or *Notch3* siRNA (siNotch3) for 48 h and activated with LPS for the indicated times. Quantitation of Western blots is shown. Means ± SD of at least three independent experiments are shown. One-way ANOVA/Bonferroni’s post-tests were performed. Statistical significance was determined at the level of *p* < 0.05. (**D**) Analysis of luciferase activity in Raw 264.7 cells transiently transfected with an NF-κB luciferase reporter (NF-κB-luc) and a *Notch3* expression vector (+ *Notch3*) or their corresponding empty vectors. One day after transfection, cells were treated for one hour with the p38 inhibitor SB203580 and then stimulated with LPS (100 ng/ml) for the indicated times. The means ± SD of three independent experiments are shown. One-way ANOVA/Bonferroni’s post-tests were performed. *p < 0.05 compared to control, untreated cells. ^#^*p* < 0.05 compared to control, LPS-treated cells. ^§^*p* < 0.05 compared to *Notch3*-transfected, SB203580 untreated cells.

Classical induction of NF-κB by TLR-4 signaling involves the activation of the IκB kinase complex mediated by the phosphorylation of the IKKβ subunit in Ser181^30^. We evaluated IKKβ phosphorylation in both control and *Notch3* siRNA-transfected macrophages stimulated with LPS for different times. As Fig. 7A shows, regardless of *Notch3* expression levels, IKKβ phosphorylation levels were comparable, indicating that activation of the IκB kinase complex in macrophages is similar either in the presence or in the absence of *Notch3*, and suggesting that this signaling process is not responsible for the effect that NOTCH3 exerts on NF-κB activity.

Multiple studies have shown that p38 MAP kinase signaling is necessary for the activation of NF-κB in response to different stimuli, including LPS^31,32^. We therefore analyzed whether NOTCH3 exerted any effect on the activation of p38 in mouse peritoneal macrophages activated with LPS. As shown in Fig. 7A, stimulation with LPS induced a strong p38 phosphorylation in control cells; however, when *Notch3* expression was decreased by siRNA, a specific inhibition of p38 phosphorylation was observed. This observation is sustained by the lower level of phospho-p38 in LPS-induced *Notch3*^*−/−*^ macrophages (Fig. 7B).

Different studies have described that p38 MAPK is involved in the phosphorylation of nuclear RelA/p65 at Ser276 through the modulation of the enzyme MSK1, resulting in its complete activation^31,32^. Besides, another residue phosphorylated by the IκB kinase complex, Ser536, allows the translocation of NF-κB to the nucleus and its binding to target genes^33^. We therefore decided to analyze whether inhibition of NOTCH3 expression in LPS-activated macrophages exerted any effect on RelA/p65 Ser276 and/or Ser536 phosphorylation. As shown in Fig. 7C, inhibition of NOTCH3 expression caused a reduction in p65 Ser276 phosphorylation in response to LPS, but no significant effect on p65 Ser536 phosphorylation was observed. These results show that NOTCH3 regulates the activation of p65, mainly by promoting phosphorylation in its Ser276 residue, probably through p38 MAPK, in macrophages activated with LPS.

To confirm the role of p38 MAPK in the increased activity of NF-κB/p65 induced by NOTCH3, we examined whether p38 inhibition could affect NF-κB activation triggered by LPS in a NOTCH3-dependent manner. We analyzed the activity of an NF-κB reporter gene transfected in control (Raw) or *Notch3*-overexpressing Raw 264.7 cells (Raw-Notch3) activated with LPS in the presence or in the absence of the specific p38α/β kinase inhibitor SB203580. As Fig. 7D shows, the data obtained confirmed that NOTCH3 overexpression increased basal and LPS-induced NF-κB transcriptional activity. However, treatment with SB203580 reversed this effect. These results suggest that NOTCH3-mediated activation of p38 enhances the transcriptional activity of NF-κB.

## Discussion

In the last decade, multiple research studies have shown the relevant role of NOTCH signaling in the differentiation and activation of myeloid cells, especially macrophages^34^, and its involvement in the development of pro-inflammatory diseases^3^. In this work, we have characterized the molecular events that take place in NOTCH signaling in TLR-activated macrophages, identifying the ligands and proteases implicated in the process. More importantly, we have revealed a prominent role of NOTCH3 in macrophage pro-inflammatory gene expression through increased p38-dependent NF-κB activation.

Niche-derived ligands are essential for the activation of NOTCH receptors. In this work we reveal that both DLL1 and DLL4 are relevant in basal and TLR-activated macrophage NOTCH signaling. These results agree with previous results showing that DLL4 was the most effective ligand for inducing NOTCH signaling and the expression of pro-inflammatory genes^25,35^, and in the development of inflammation in the cardiovascular system and other organs^35,36^. On the contrary, JAGGED1 showed a clear inhibitory effect, similar to that described in other cellular models, such as cancer cells, where JAGGED1 interaction with NOTCH3 inhibits the apoptosis induced by this receptor^36^. Nevertheless, in a model of atherosclerosis, endothelial cells promote inflammatory leucocyte recruitment and inflammation through JAGGED1-RBPJ signaling^37^. Glycosylated NOTCH receptors have higher affinity for DLL than for JAGGED ligands^38^. It is therefore possible that a stronger glycosylation of NOTCH receptor in the course of macrophage activation would allow a better interaction with DLL ligands.

Using functional studies in mammalian cells, different groups have established that the metalloprotease ADAM10, but not ADAM17, is essential for ligand-induced NOTCH1 extracellular cleavage, both in cellular models and in vivo^18,19^. Nevertheless, increasing evidence shows that in ligand-independent NOTCH1 signaling, as is the case with mutated NOTCH1 receptors in T-ALL, other proteases may be involved, in particular ADAM17^39,40^. In this work, we analyzed the ADAM10/17 NOTCH signaling dependence after TLR-4 macrophage activation and found that this process is mainly dependent on ADAM10, although a small but significant effect of ADAM17 was detected when CBF activity was evaluated in the absence of ligands. The effect of ADAM17 did not affect NOTCH1 activation, which seems to depend exclusively on ADAM10. New approaches will be needed to characterize the discrete effect of ADAM17 on activated macrophages.

Although the different NOTCH receptors play distinct roles in multiple differentiation processes, as evidenced by the different phenotypes observed in animals with targeted deletion of each receptor^13,15,41^, many redundant effects have also been described^42^. Our main objective was to analyze and characterize the individual contribution of each NOTCH receptor to macrophage activation. We observed that NOTCH1, 2 and 3 participate in the increased NOTCH signaling observed in macrophages after TLR-4 activation. Interestingly, NOTCH3 and, to a lesser extend NOTCH1, showed a clear specificity for NF-κB activation. An implication of NOTCH3 in NF-κB activation has been previously described in thymocytes and T lymphoma cells derived from transgenic mice overexpressing the NOTCH3 intracellular domain (NICD3)^43^.

The specific activity of NOTCH3 has been related to structural differences with respect to NOTCH1 and 2, particularly within the transactivation region. Those differences pertain to the weak transactivation activity of the NOTCH3 intracellular domain when compared to that of NOTCH1 and NOTCH2^44^. These differences could also possibly explain the different capacity of NICD3 to recruit coactivators and/or corepressors, as well as to undergo particular conformational changes^45^. However, in some instances, the signaling induced by NOTCH1 and NOTCH3 are very similar. In T-cell acute lymphoblastic leukemia, the intracellular domains of NOTCH1 and NOTCH3 are effectively interchangeable in their ability to drive the expression of downstream target genes that support the aberrant cell growth. Indeed, a comparative analysis of the genomic landscape of NOTCH binding sites and of the transcriptional response to activated NOTCH shows that the oncogenomic effects of NOTCH3 and NOTCH1 in T-ALL cells are highly overlapping^42^.

NOTCH3 displays a more restricted tissue distribution than NOTCH1 and NOTCH2, being expressed predominantly in vascular smooth muscle^46^, central nervous system^47^, some thymocyte subsets^48^ and regulatory T cells^49^. Previous results showed that NOTCH3 expression selectively increases during macrophage differentiation, and that *Notch3* knockdown during this process decreased the transcription of proinflammatory genes, such as iNOS^50^. The data presented here are in complete agreement with these results and show that NOTCH3 is the main NOTCH receptor expressed by resting macrophages, and that LPS activation induces a rapid increase in NOTCH3 levels, and increased translocation of NICD3 to the nucleus. Crystallography studies have shown that the conformation of the NOTCH3 negative regulatory region (NRR) is less stable than that of NOTCH1 and NOTCH2, which makes it easier to cleave by ADAM proteases^51^. This rapid NOTCH3 activation could explain the induction of *Hes1* observed at relatively short times (2 h), even when the rest of NOTCH receptors have not yet been synthesized. Besides, it has recently been described that NOTCH3 can be clearly activated independently of the interaction with canonical ligands and mediate the growth of basal breast cancer cells^42,52^, arguing for a new mechanism of NOTCH3 stimulation that could also be involved in macrophage activation.

Our work confirms and highlights the relevance of NOTCH3 expression and signaling in pro-inflammatory macrophage activation and identified its prominent and specific role in the activation of NF-κB. A positive regulation between NOTCH and NF-κB signaling pathway has been described previously in macrophages isolated from patients with atherosclerosis. In those patients, and in contrast with our results, NOTCH1 appears to play a more prominent role than those of NOTCH2 and NOTCH3 in the regulation of NF-κB signaling pathway and in the induction of pro-inflammatory gene expression^53^. NOTCH3 is also induced in kidney epithelium after ischemia/reperfusion injury in a process mediated by NF-κB binding to the *Notch3* promoter. This induction correlates with a sustained pro-inflammatory response that leads to a rapid deterioration of renal function^54^.

**Figure 8:**
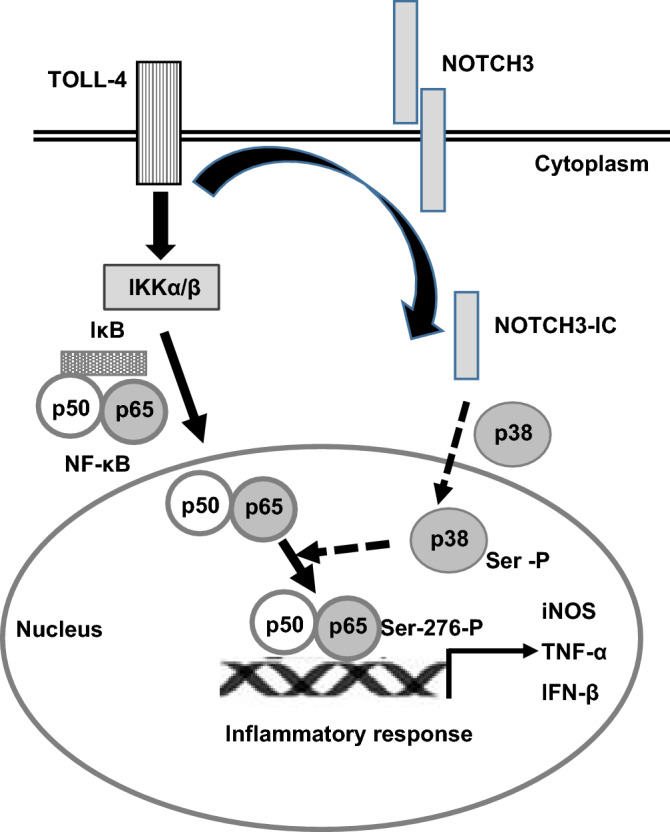
Picture representing the mode of action of the NOTCH3 receptor in the process of macrophage activation by TLR-4. TLR-4 signaling induces IKKα/β activation, which favors IkB phosphorylation and degradation, allowing NF-κB translocation to the nucleus. TLR-4 signaling induces as well NOTCH3 activation, which in turn modulates the phosphorylation and activation of p38 MAP kinase, which favors p65 phosphorylation on Ser276. Phosphorylation of p65 on Ser276 increases the transcriptional activity of NF-κB and is essential for the expression of a set of NF-κB-activated genes, such as *TNFα*, *iNOS*, *IL-6* or *IFN*β.

We previously described that NOTCH1 signaling increases NF-κB activity in LPS-activated macrophages. This process seems to be mediated by a higher IKK-β activation, which favors phosphorylation and degradation of IκBα and the phosphorylation of p65 in Ser536^25^. In contrast to what happens with NOTCH1, in this study we have shown that NOTCH3 does not affect IKK-β phosphorylation, but modulates p38 kinase activity, which, in turn, favors p65 phosphorylation at Ser276 (see the scheme in Fig. 8). The regulation of p38 through NOTCH3 has also been observed in smooth muscle cells of the arterial vasculature^55^, and a deficient p38 phosphorylation has also been described in RBPJ-deficient macrophages^34^. Nevertheless, the mechanism by which NOTCH3 modulates the activation of p38 is still unknown. Phosphorylation of p65 on Ser276 increases the transcriptional activity of NF-κB by favoring its binding capacity to CBP/p300 coactivators, and in this way enhances NF-κB transactivation ability^33,56,57^. Phosphorylation of p65 on Ser276 is essential for the expression of a set of NF-κB-activated genes^57^, including *TNF*α. Previous results have also shown the dependence of *iNOS*, *IL-6* and *IFN*β expression on p38 activity^58^. All the above-mentioned results agree with our own, which show that *Notch3*^*−/−*^ macrophages or macrophages transfected with *Notch3* siRNA have lower NOTCH3 expression levels, and also express lower levels of pro-inflammatory cytokines. Phosphorylation of p65 both on Ser276 and Ser536 is essential for NF-κB activation, through pathways integrated in the pro-inflammatory macrophage activation by TLR-4. Our results indicate that receptors NOTCH1 and NOTCH3 act together to reach maximal NF-κB activation. This could explain their synergistic effects on the activity of the NF-κB reporter gene observed in Raw 267.4 cells.

Our results also indicate that a crosstalk between NOTCH3 and NOTCH1 takes place in the course of macrophage activation by TLR-4 receptors. A transient increase in NOTCH3 expression is observed in the first hours after activation, followed by a gradual fall, coincident with an intense increase in NOTCH1 expression. Thus, it appears that NOTCH3 is replaced by NOTCH1 after macrophage activation. A similar interplay between NOTCH1 and NOTCH3 seems to coordinate esophageal squamous cell differentiation through a CSL-dependent transcriptional network^59^, with NOTCH1 self-regulating its own transcription, as previously reported^60^. NOTCH1 is also required for *Notch3* induction, and the effect is ascribed to the presence of NICD1 and CSL binding sites on the second intron of the *Notch3* gene^60,61^. However, our results do not agree with those observed in squamous cells, as we detected increased expression of *Notch3* in *Notch1*^*−/−*^ macrophages^59^. Interestingly, this effect is coincident with the increased *Zeb1* expression observed in TLR-4-activated macrophages. ZEB1 is a transcription factor acting as a negative regulator of NOTCH1 target genes, including *Notch3*^27^, which is induced in a NOTCH1-dependent manner. ZEB1 enhances the NF-κB/iNOS signal pathway in osteosarcoma cells^62^. It also controls macrophage chemotaxis and migration, and promotes mobilization of pro-inflammatory monocytes into inflammatory and infection foci, and tumors^63^. So, it is possible that ZEB1 expression could be another mechanism by which NOTCH1 modulates macrophage activation. New experiments are granted to explore and confirm or not this hypothesis.

Our results suggest that NOTCH3 is the main NOTCH receptor controlling the expression of NF-κB-dependent pro-inflammatory genes in the first hours after TLR-4 activation, but NOTCH1 takes control later on. In other cellular contexts, it has also been observed that NOTCH1 and NOTCH3 signaling is very similar, for example in acute T-cell lymphoblastic leukemia, in which the activation of both NOTCH1 and NOTCH3 has the ability to induce T-ALL^64^. As happens with other members of the NOTCH family, dysregulation of NOTCH3 signaling underlies human pathologies. Constitutive activation of NOTCH3 have been related with T-cell leukemogenesis, as previously commented^65–67^. On the contrary, NOTCH3 mutations abrogating proper receptor folding and signaling are associated with CADASIL, a disease that causes progressive hypertrophy of the smooth muscle cells in blood vessels. CADASIL is caused by cysteine-altering pathogenic variants in epidermal growth factor-like repeat (EGFr) domains of the NOTCH3 protein, and is characterized by the presence of subcortical infarcts and leukoencephalopathy^68^. Similarly, *Notch3* knockout mice are more susceptible to ischemic stroke^69^. Further studies in CADASIL patients or *Notch3* knockout mice could reinforce our knowledge of the role of NOTCH3 in inflammation.

## Materials and methods

### Mice

We generated mice with myeloid-specific deletions of *Notch1* and/or *Notch2* by crossing *Notch1*^*flox/flox*^, *Notch2*^*flox/flox*^ animals to animals with a lysozyme promoter-driven Cre transgene on a C57/BL6 background (Jackson Laboratories). All procedures carried out with mice followed the European and Spanish regulations and were approved by the Ethics in Animal Care Committee of the University of Castilla-La Mancha. Mice were genotyped by PCR, using tail DNA obtained with GeneJET Genomic Purification Kit (Fermentas) with the oligonucleotides *Genot Cre* + , *Genot Cre-*, *Genot Notch1* and *Genot Notch2* (Table 1). Notch3 deficient mice (Notch3^*−/−*^) were kindly provided by Dr. A. Joutel to I. Screpanti^41^.

**Table 1:** Oligonucleotides used for PCR.

Gene	Forward primer	Reverse primer
Genot Cre +	5-’TTA CAG TCG GCC AGG CTG AC-3´	5´-CTT GGG CTG CCA GAA TTT CTC-3´
Genot Cre−	5´-CCC AGA AAT GCC AGA TTA CG-3´	5´-CTT GGG CTG CCA GAA TTT CTC-3´
Genot Notch1	5´-TGC CCT TTC CTT AAA AGT GG-3´	5´-GCC TAC TCC GAC ACC AAT A-3´
Genot Notch2	5´-TAG GAA GCA GCT CAG CTC ACA G-3´	5´-ATA ACG CTA AAC GTG CAC TGG AG-3´
mP0	5′-GAA TCG CTC CTG CAG CAA AG-3’	5′-CCA GGG TCT CAT CCG CAT T-3’
mFurina	5′-GAG CAC AAC AGC AGC ATC CAC ATT-3’	5′-TGA TGG CAC AAC TCC ACT GAT CCT-3’
mNotch1	5´-AGG CAA GCA ACA GGA GTA AG-3´	5´-GTG TCT ACG CTC GGC TAA TG-3´
mNotch2	5´-ATG AAC TGC CAG ATG TCC-3´	5´-TGT CAG GCA AAA TCA GAA AG-3´
mNotch3	5´-AAG CGC GTC CTG GCA TTG TCT-3´	5´-CCG CAG GGG CAG CAG TGG T-3´
mNotch4	5-’AAA GGT GTC GTC CTC TCC ACC-3’	5′-CAA ATT CTG TGC CAG GCA-3’
hNotch1	5′-CAC CGG GAC ACC GTT ATT T-3’	5′-GGC AAGGAGAAG AGG AA-3’
hNotch2	5′-ACC TGC TCT TCA GCT ACA ATA TC-3’	5′-CTC TCA CCC AGT CTT GGA ATA AA-3’
hNotch3	5- ‘CCG GAG AGG AAT CGA GAA TTA C-3’	5′-GAG TGG CCA TCA GAC ACT ATA C-3’
hNotch4	5′-TCG CCC TCC TCC AGA TAT T-3’	5′-CTC CTA AGC CAC ACA CAG ATA C-3’
mzeb1	5′-CTG GGA GGT CAG TAT GGA AAT C-3’	5′-CTC TCT GCT GGC TCA ATG-3’
mIfn	5′-GAA GAG ACG AGC TGA ACC TAAC-3’	5′-CTG CTC TAT CTG CAC TCC ATA C-3’
mNos2	5′- CTG CTG GTG GTG ACA AGC ACA TTT-3'	5′-ATG TCA TGA GCA AAG GCG CAG AAC-3’

### Cells and reagents

Peritoneal macrophages were isolated as previously described^2^ from two-month-old *Notch1* and/or *Notch2* KO or WT male mice, or from ten week-old Notch3^*−/−*^ mice, 4 days after i.p. injection of 2 mL 3% sterile thioglycolate broth (w/v in water, Gibco). Elicited macrophages were seeded at 1 × 10^5^ cells/cm^2^ in complete RPMI 1,640 medium (supplemented with 10% FBS -Fetal Bovine Serum-, 2 mM L-glutamine, and 1% penicillin–streptomycin, all from Lonza), and incubated overnight in complete RPMI medium supplemented with 2% FBS, preceding activation with either 100 ng/mL LPS from *Salmonella typhimurium* (Sigma-Aldrich), 200 ng/ml of poly I:C (Amersham Bioscience), 5 μg/ml of lipoteichoic acid from *Staphylococcus aureus* (InvivoGen) and/or 20 U/ml of interferon-γ, and/or 1 µM Dexametaxone. Activation was verified by the Griess test.

To limit NOTCH receptor activation, inhibitors for each of the enzymes responsible for proteolytic activation were used: for ADAM10, 5 μM GI254023X (Aobious, Gloucester, USA); for ADAM17, 5 μM GW280264X (Aobious, Gloucester, USA); and for γ-secretase, 10 μM DAPT (Calbiochem). To induce ligand-independent NOTCH receptor activation, cells were washed twice with phosphate-buffered saline (PBS; Lonza) and then incubated in 5 mM EDTA (Panreac) in PBS or PBS alone for 10 min. at 37 °C, as previously described^70^. To inhibit protein synthesis, 2.5 µg/ml cycloheximide (CHX) was used (Roche, Manheim, Germany) for the times indicated.

Raw 264.7 cells (ATCC No TIB-71) and 3T3-L1 cells (ATCC No CL-173), were subcultured at 6–8 × 10^4^ cells/cm^2^ in DMEM medium (Lonza) supplemented with 10% FBS , 2 mM L-glutamine and 100 U/ml penicillin–streptomycin, and incubated overnight in complete DMEM supplemented with 2% FBS, preceding activation with 100 ng/mL LPS from *Salmonella typhimurium*, and/or 20 U/ml interferon-γ. Activation was verified by the Griess test.

Human monocytes were isolated from blood of anonymous healthy donors of the Blood Bank of the University Hospital of Albacete, Spain. The Ethics Committee for Human Research of the University Hospital of Albacete approved the study and informed consent procedures (Approval Number 4-11/08), in accordance with the tenets of the Declaration of Helsinki and following the European and Spanish regulations. Informed written consent was obtained from all the participants and was recorded by the staff involved in the study. Samples were centrifuged on Ficoll-PaqueTM PLUS (Amersham Biosciences), following standard protocols^71^ and cultured in complete DMEM medium supplemented or not with 100 ng/mL LPS for 24 h.

### Cell transfections

For transient transfections, 2.5 × 10^5^ Raw 264.7 cells per well were seeded in triplicate on 12-well plates and transfected on the following day with Lipofectamine 2000 (Invitrogen), according to the manufacturer’s recommendations, using OPTI-MEM medium (Gibco) without supplements and 1.25 µg of total EndoFree plasmid DNA per well. The reporter plasmids pNF-κB-luc, pCBF-luc, used to detect NF-κB- and NOTCH-dependent transcription activities, respectively, have been previously described^72^. pRLTK Renilla-expressing vector (Promega) was used as a control for transfection efficiency. pLNCX2 (empty vector), pLNCX2-Delta4 (murine *Dll44* expression vector), Sh-Control (SA Bioscience, 20070705-2), murine Sh-Notch1 (SA Bioscience, KM04747N), Sh-Control (pGeneClip neomycin empty vector, Qiagen, DC24-1), murine Sh-Notch2 (Qiagen, 201204125), murine Sh-Notch3 (Qiagen, 201206045), murine Sh-Notch4 (Qiagen, 201204121), murine Sh-Adam10 (Qiagen, 200702), murine Sh-Adam17 (Qiagen, 200712273), PCD2 (empty vector) and/or PCD2-Notch1 (full-length murine *Notch1* expression vector)^73^ were used together with the reporters. Cells were stimulated with 100 ng/mL LPS for 24 h after being transfected. Luciferase and Renilla activities were measured by using the Dual Luciferase Reporter Assay System (Promega) in a Sirius luminometer (Berthold) following the manufacturer’s recommendations.

For stable transfections, 3 × 10^6^ Raw 264.7 cells were seeded on 60 mm plates 24 h before transfection with 5 µg plasmid DNA. Cells were selected with 300 μg/mL G418 (Sigma) for two weeks.

### si RNA silencing

Peritoneal macrophages were seeded on six-well plates to a cell density 1.5 × 10^6^ cells per well. The following day, macrophages were transfected with 50 nM murine siRNAs anti-Notch3 *(Dharmacon*) or the corresponding scrambled siRNA control (*Dharmacon*), using a Lipofectamine RNAiMAX reagent (Invitrogen), following the manufacturer’s recommendations. Cells were analyzed 72 h after transfection.

### Co-culture assays

Mouse OP9 cells stably expressing human Dll1, Dll4 or Jagged1 or the parenteral line were a kind gift of M.J. Toribio's laboratory and have been described previously^74^. Human and mouse Notch ligands show very high homology, especially in their extracellular domain: Jagged1 (96% identity, 98% similarity), Dll1 (88% identity, 94.5% similarity) and Dll4 (86% identity, 91.4% similarity) (The EMBL-EBI search and sequence analysis tools APIs in 2019). On the day prior to the assay, OP9 cells were seeded in 24-well plates at a density of 30,000 cells per well and maintained in α-MEM medium lacking ribonucleotides and deoxyribonucleosides, with 2.2 g/L sodium bicarbonate (Gibco), supplemented with 20% FBS, 2 nM L-glutamine, 100 U/ml penicillin and streptomycin.

On the same day, Raw 264.7 cells were seeded in 60 mm tissue culture dishes at a density of 3 × 10^6^ cells/dish and transiently transfected with ADAM10 or ADAM17 shRNAs (Qiagen) and the reporter plasmid pRBP-J-luc to detect NOTCH signaling as described above. 24 h after transfection, Raw 264.7 cells were co-cultured with the OP9 cells seeded the day before. Once Raw 267.4 cells had adhered to the OP9 cell monolayer, they were stimulated with 100 ng/mL LPS for 24 h. Lysates were collected 24 h post-activation and assayed for luciferase activity by using the Dual-Luciferase Assay kit (Promega).

### Protein extracts and Western blot analysis

To harvest the cells, they were washed with ice-cold PBS, scraped off the dishes, and finally they were collected by centrifugation. After RIPA lysis buffer resuspension (25 mM Hepes, pH 7.5; 1.5 mM MgCl_2_; 0.2 mM EDTA; 1% Triton X-100; 0.3 M NaCl; 20 mM β-Glycerophosphate; 0.1% SDS; 0.5% desoxycholic acid), we added a cocktail of protease inhibitors and I and II phosphatase inhibitors (Sigma), homogenized the extract for 30 min. at 4 °C and centrifuged at 8000×*g* for 15 min. Protein concentrations were determined by the Bradford method (Bio-Rad).

Protein extracts (30–40 µg) were separated by denaturing SDS-PAGE in 10% polyacrylamide gels; after the electrophoresis, proteins were transferred to nylon membranes (Hybond-C Extra, Amersham Biosciences) and processed according to the antibody suppliers’ recommendations. We used ECL (Amersham Pharmacia Biotech) for protein detection, and β-tubulin expression as a loading control for cytosolic and total protein extracts, and Laminin B for nuclear extracts.

Anti-cleaved NOTCH1 (Val1744), anti-pIKKα(Ser180)/β(Ser181), anti-p38α MAPK (L53F8), anti-pp38 MAPK (Thr180/Tyr183) (3d7), anti-NF-κB p65 (D14E12), anti-pNF-κB p65 (Ser536) (93H1), anti-IRF1(D5E4), were purchased from Cell Signaling. Anti-Notch1 (C-20), anti-Notch2 (M-20) and anti-Hes1 (H-20) from Santa Cruz; and anti-iNOS from BD. Anti-ADAM10 and anti-Notch 4 were purchased from Millipore, anti-Notch3 (Ab 23426), anti-furin (Ab3467) and anti-pNF-κB p65 (Ser276) (Ab 106129) from Abcam and anti-α-tubulin from Sigma.

### RNA and cDNA purification

Total RNA was obtained by using the RNeasy Kit (Qiagen) with DNase (Promega), according to the manufacturer's instructions. The concentrations were evaluated in a ND-1000 (NanoDrop) spectrophotometer. cDNA synthesis was performed using 1 µg of total RNA with the RevertAidH Minus First Strand cDNA Synthesis (Fermentas), following manufacturer´s recommendations^75^.

### Quantitative RT-PCR

Gene expression analysis by quantitative RT-PCR (qRT-PCR) was performed in triplicate according to the Fast SYBR Green Protocol with the StepOne real-time PCR detection system (Applied Biosystems). Specific oligonucleotides were designed with the PrimerQuest SM computer program (Integrated DNA technologies, Inc.) and are indicated in Table 1. The mRNA levels of mouse riboprotein P0^76^ or human GAPDH were used as internal controls^75^.

### Statistical analysis

The student’s unpaired *t*-test was used for statistical analyses between two groups. The statistical analysis between more than two populations was carried out using the Analysis of Variance (ANOVA). If there was statistical difference between the means, the *post-hoc* Bonferroni test at a pre-set alpha of 0.05 was used to evaluate statistical differences for each of the criteria and their interactions.

## Supplementary information

Supplementary information.
