# Supplementary material for: NOTCH3 signaling is essential for NF-κB activation in TLR-activated macrophages
Source: Sci Rep. 2020 Sep 9;10:14839. doi: 10.1038/s41598-020-71810-4 (PMC7481794; doi:10.1038/s41598-020-71810-4)
Supplement: Supplementary file 1 — Supplementary information. [file 41598_2020_71810_MOESM1_ESM.pdf]

Supplementary information

NOTCH3 signaling is essential for NF-κB activation in TLR-activated macrophages

López-López, Susana[1]; Monsalve, Eva[1]; Romero de Ávila, María José[1]; González-Gómez, Julia[2]; Hernández de León, Natalia[3]; Ruiz-Marcos, Francisco[3]; Baladrón, Victoriano[1]; Nueda, María Luisa[2]; García-León, María Jesús[4]; Screpanti, Isabella[5]; Felli, María Pía[6]; Laborda, Jorge[2]; García-Ramírez, José Javier[1*] and Díaz-Guerra, María José M.[1*]

Following are the full-length immunoblots for **figure 1B**

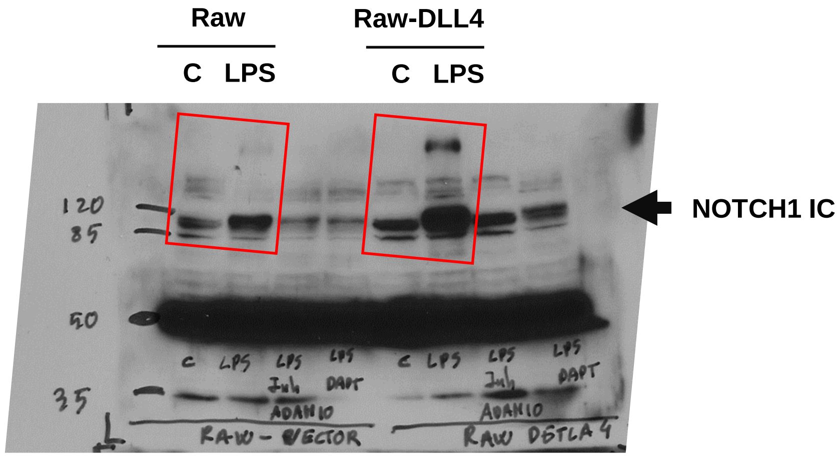

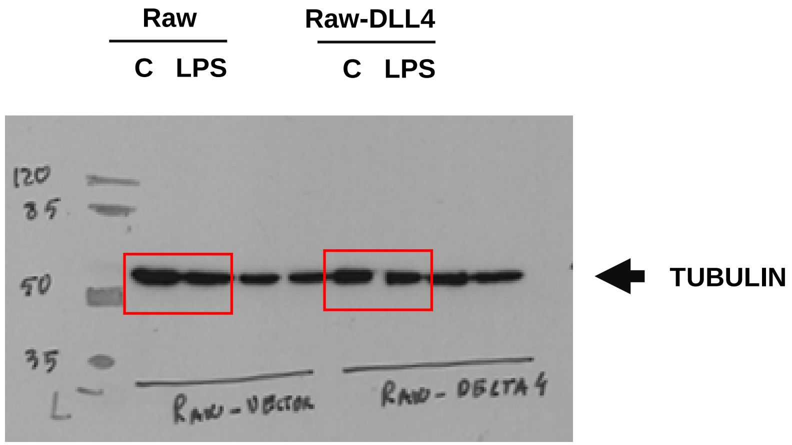

Following are the full-length immunoblots for **figure 1D**

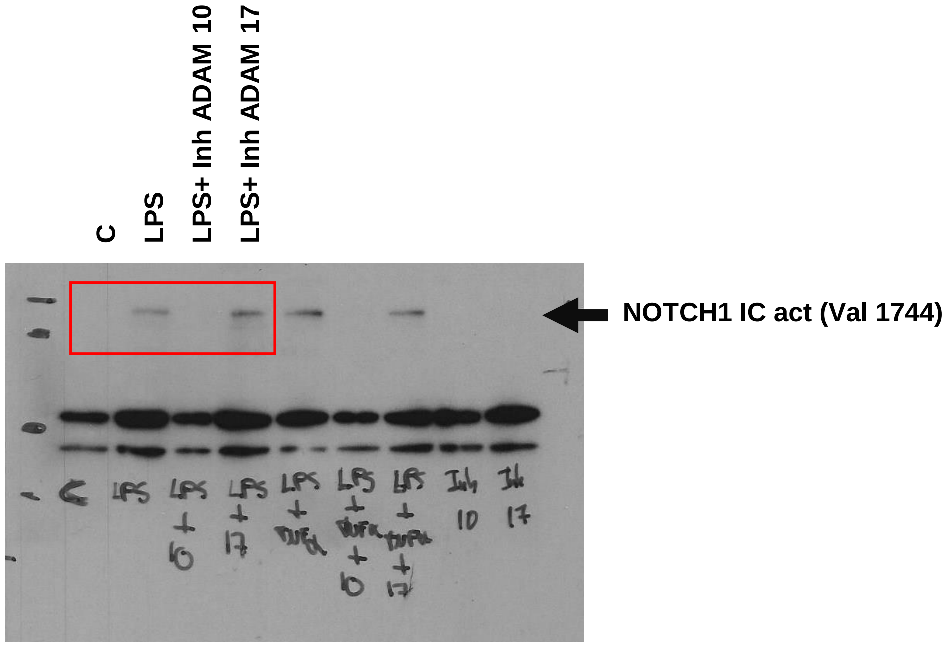

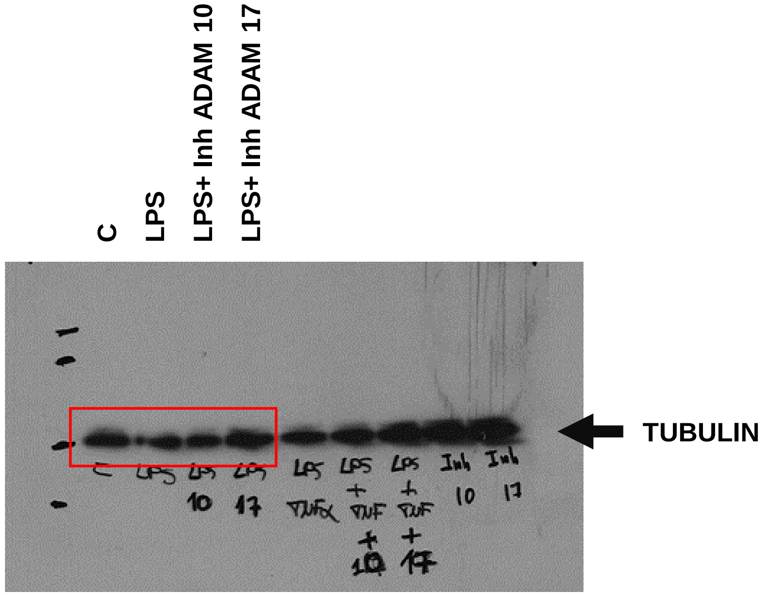

Figure 2A

Following are the full-length immunoblots for **figure 2A**

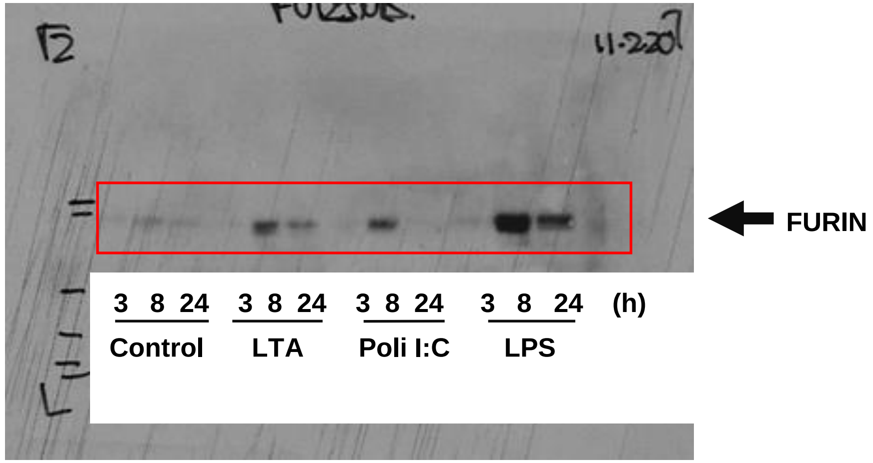

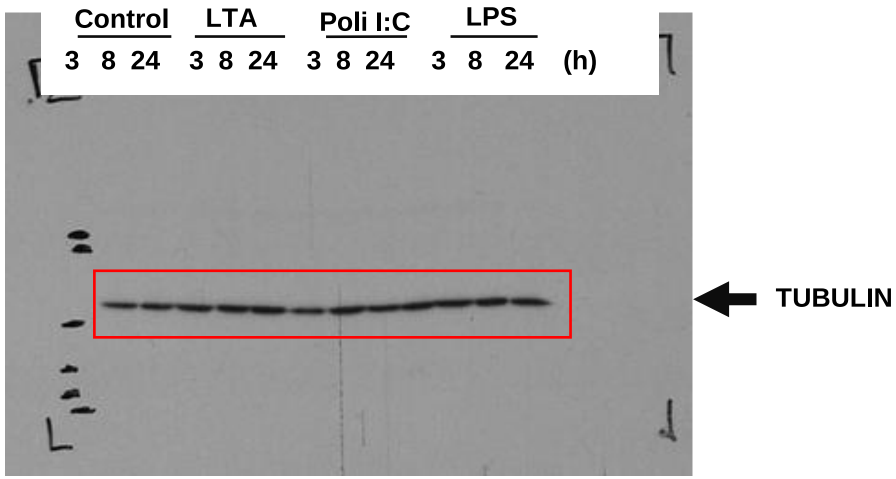

Following are the full-length immunoblots for **figure 2B**

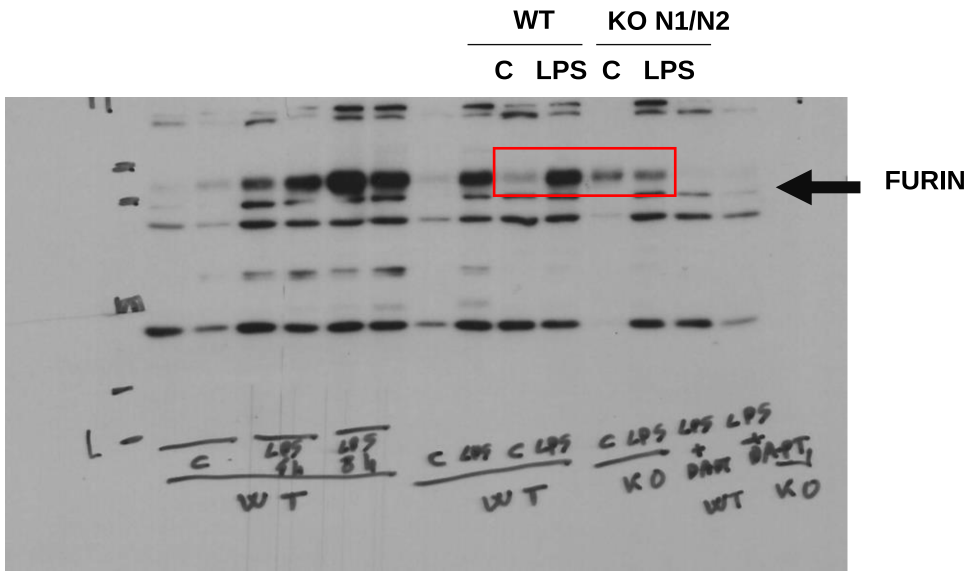

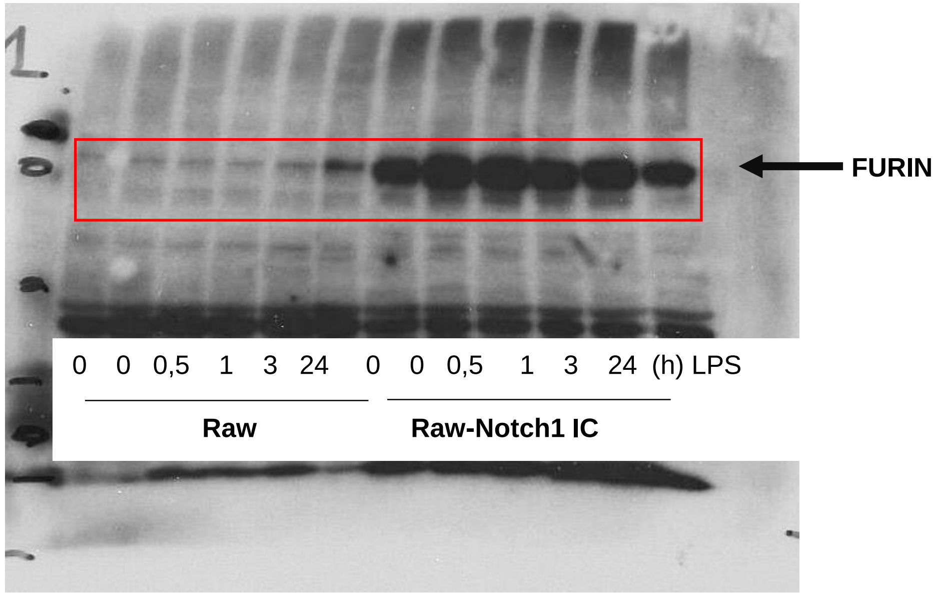

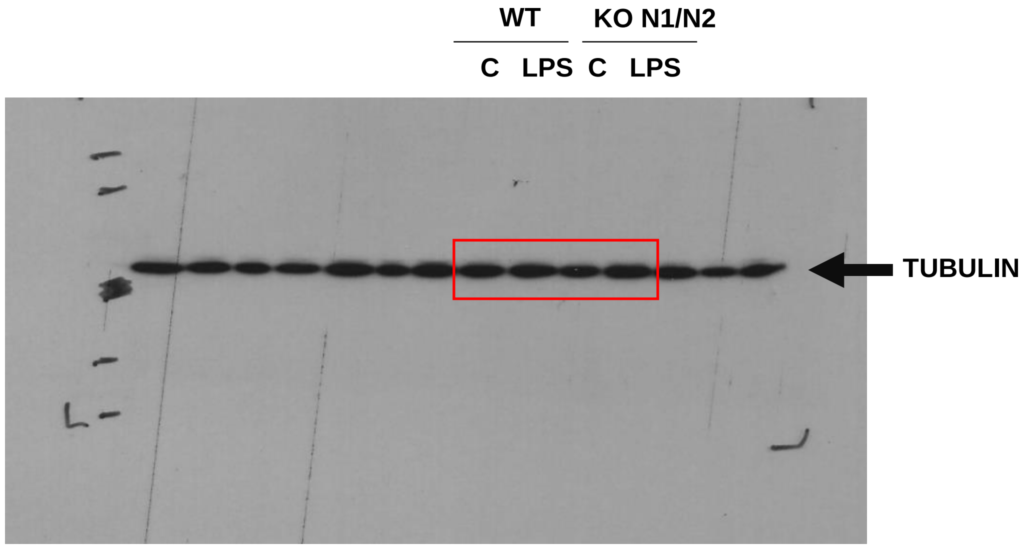

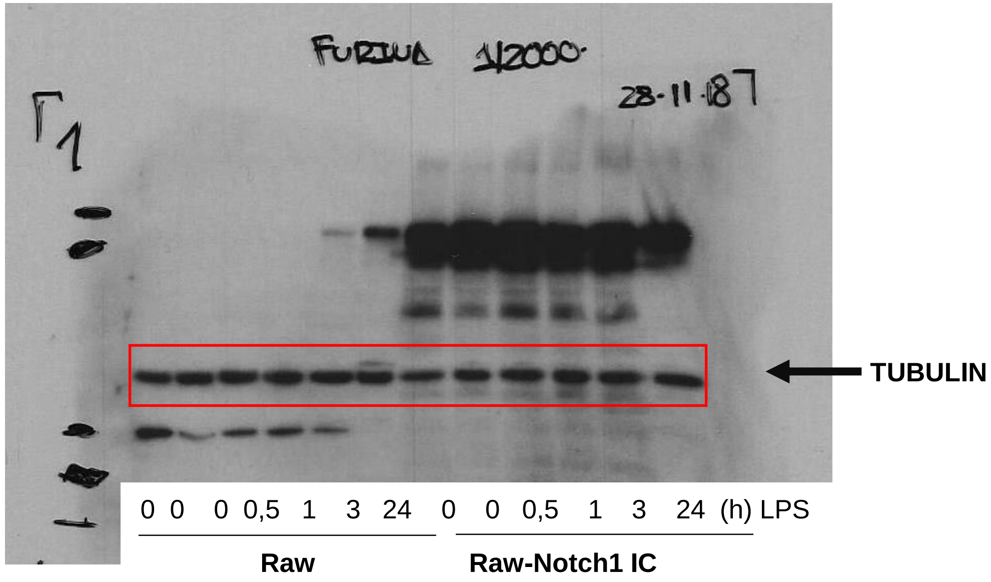

Following are the full-length immunoblots for **figure 2C**

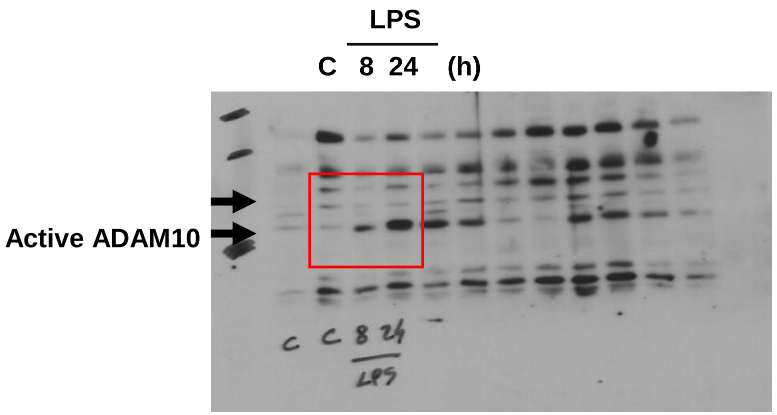

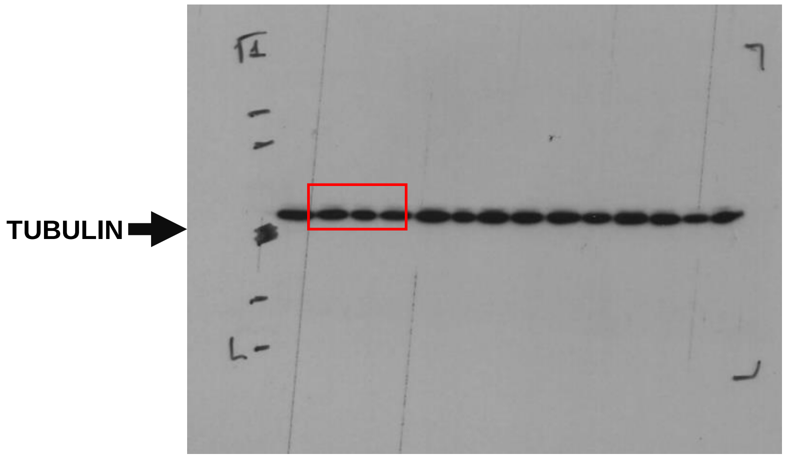

Following are the full-length immunoblots for **figure 2F**

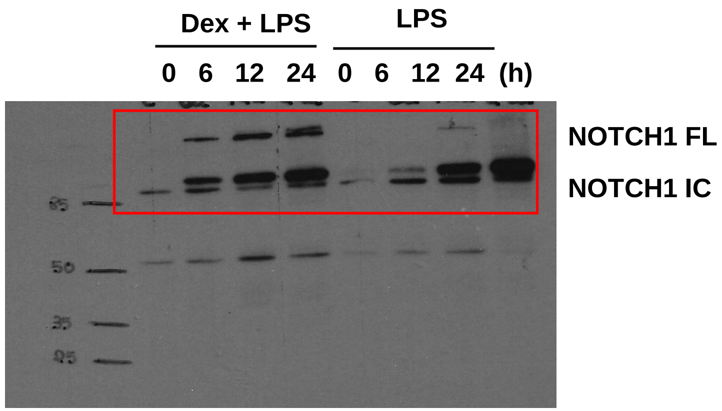

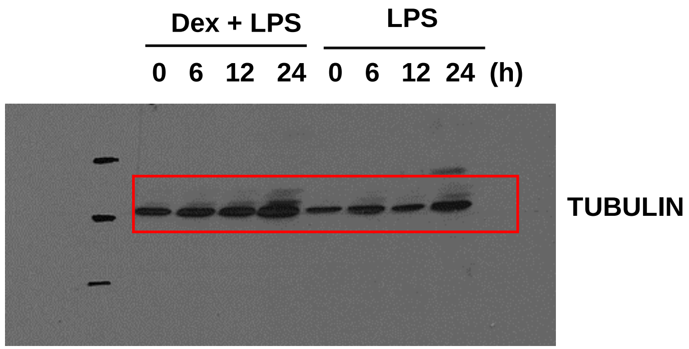

Following are the full-length immunoblots for **figure 3A**

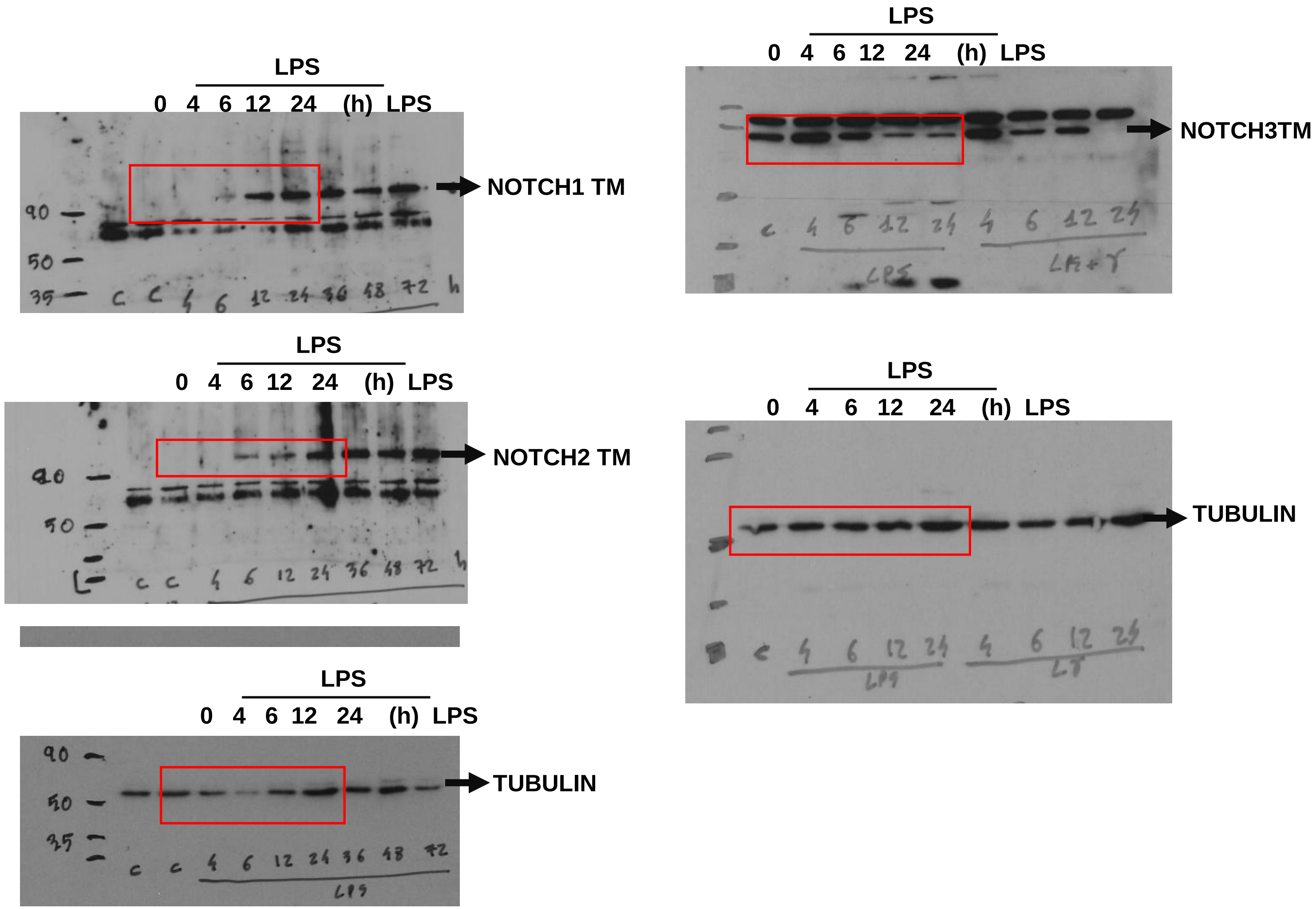

Following are the full-length immunoblots for **figure 3A**

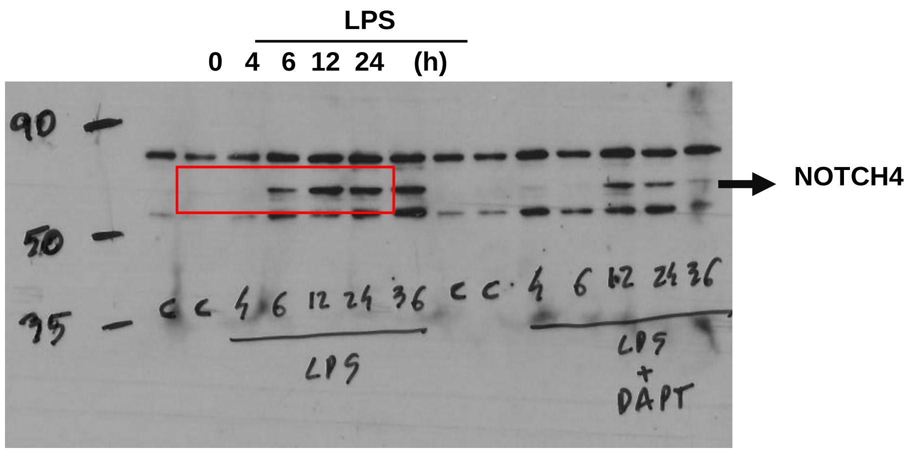

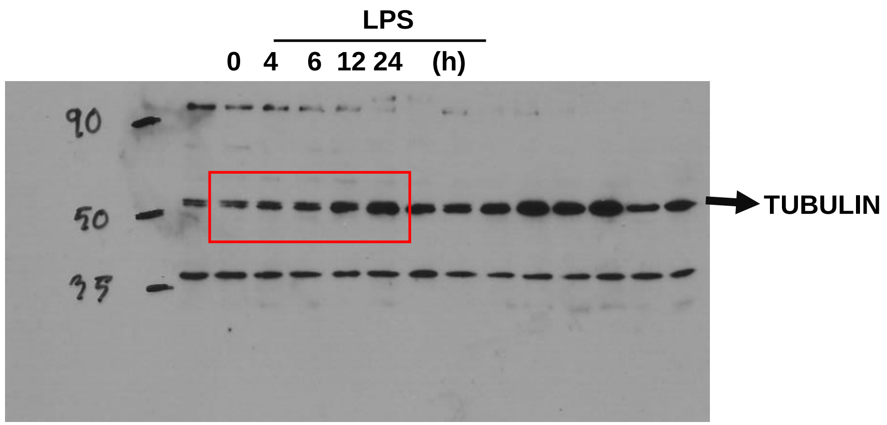

Following are the full-length immunoblots for **figure 3D**

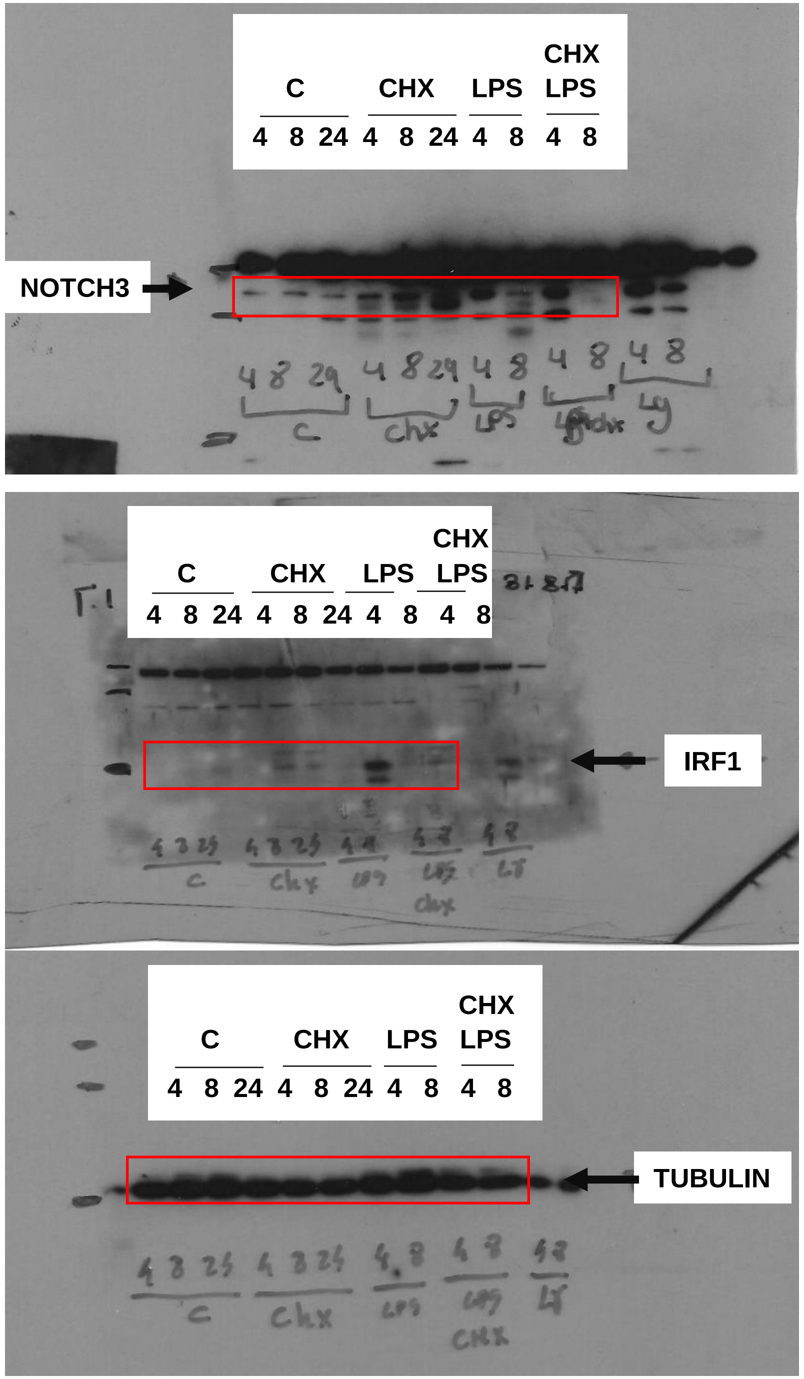

Following are the full-length immunoblots for **figure 3E**

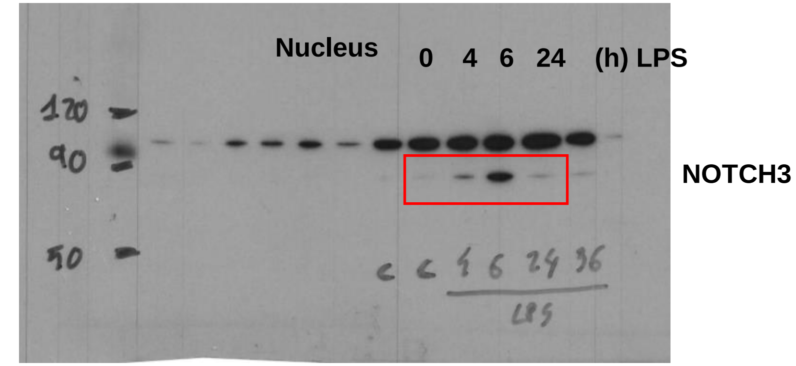

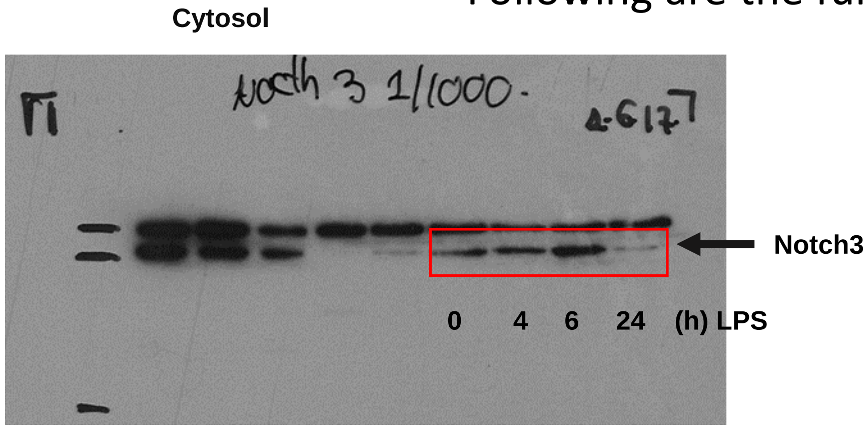

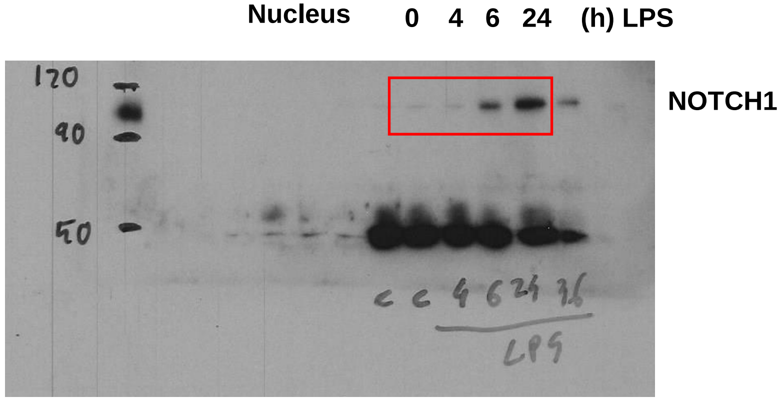

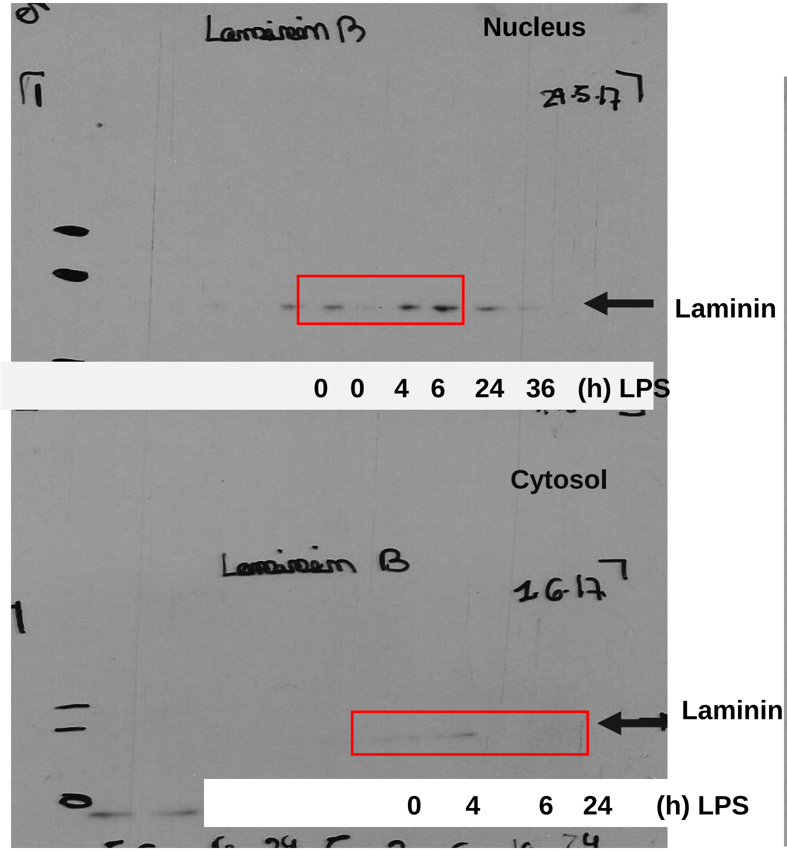

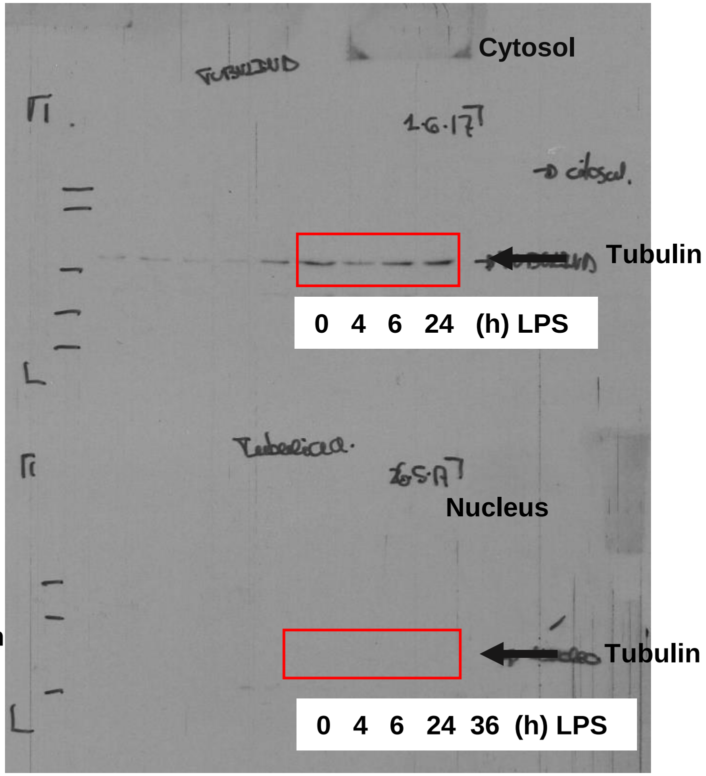

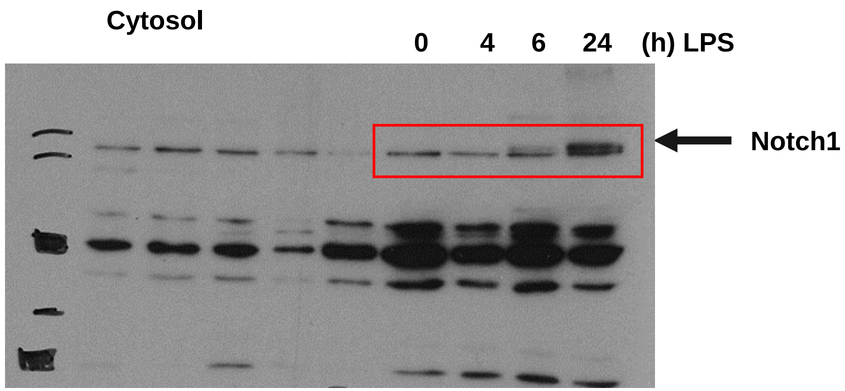

Following are the full-length immunoblots for **figure 3F**

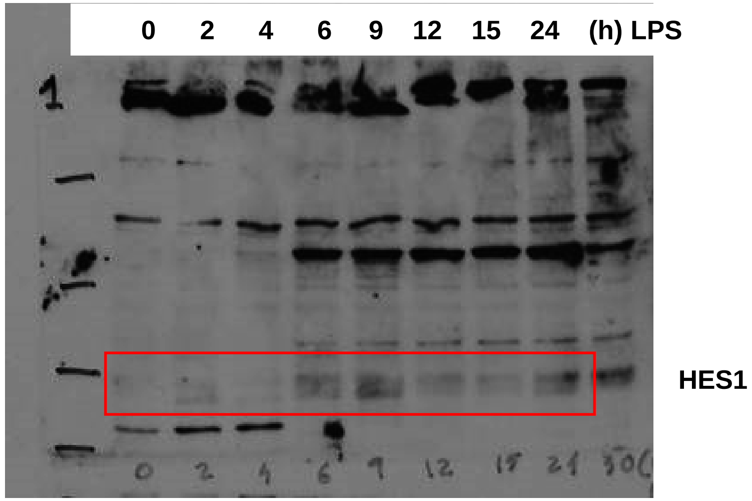

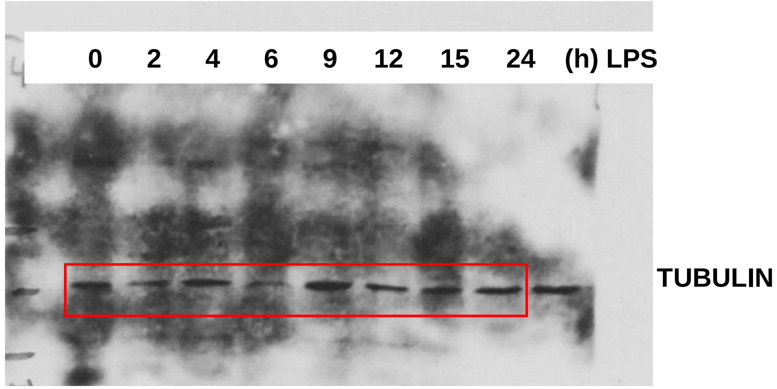

Following are the full-length immunoblots for **figure 4A**

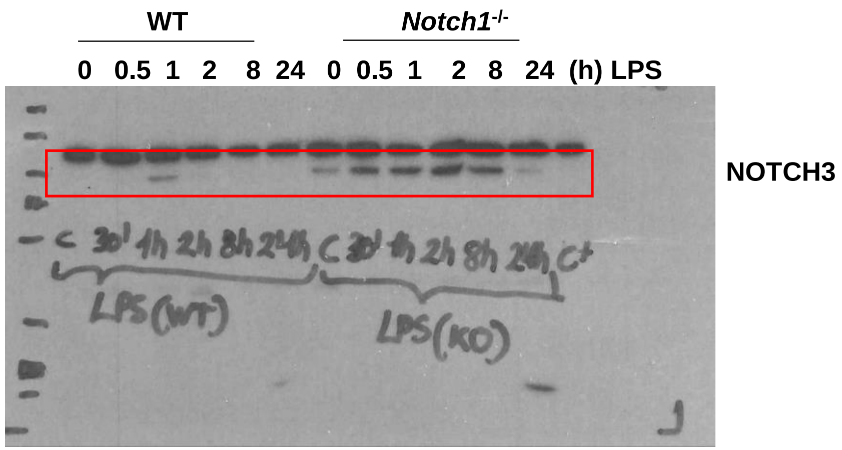

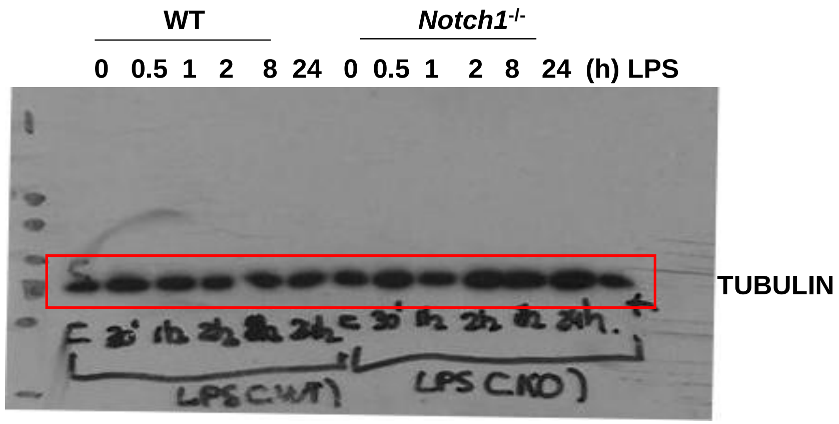

Following are the full-length immunoblots for **figure 4c**

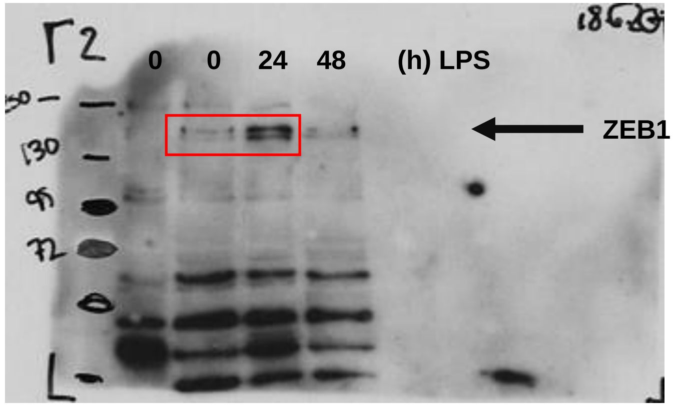

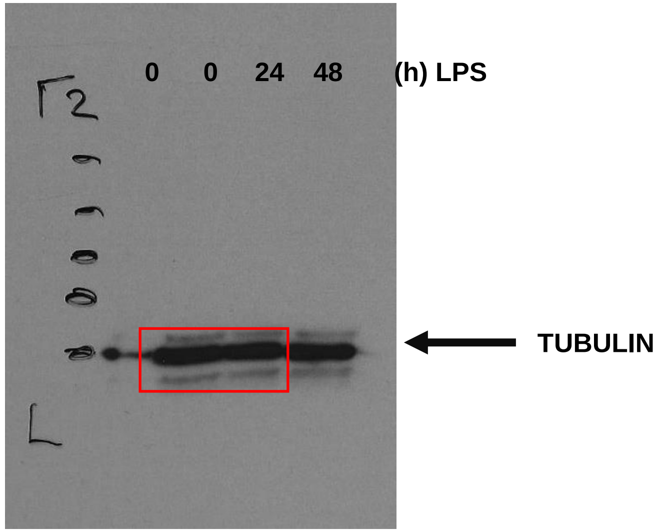

Following are the full-length immunoblots for **figure 4E**

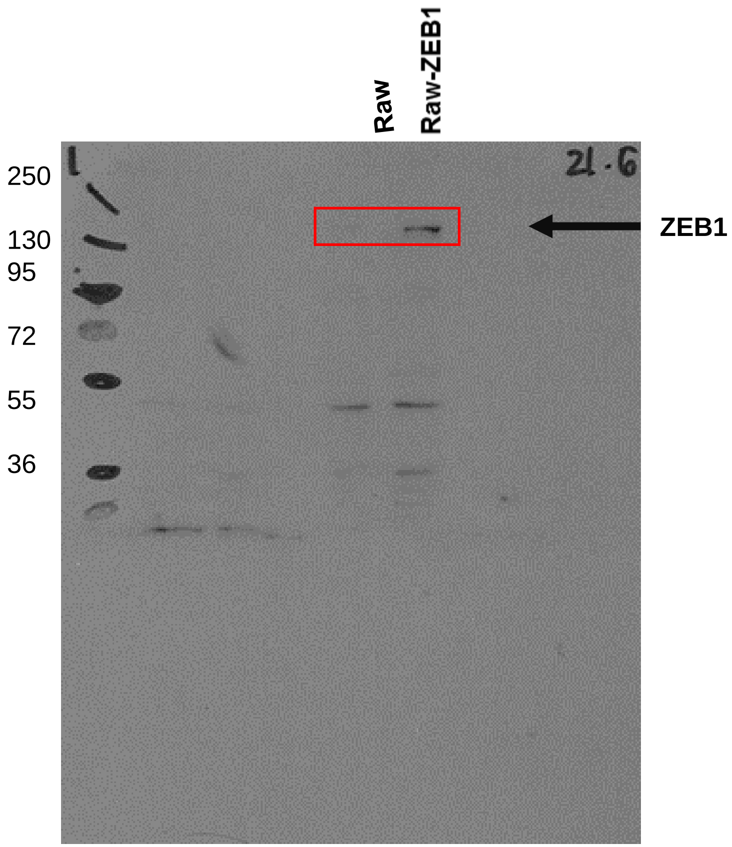

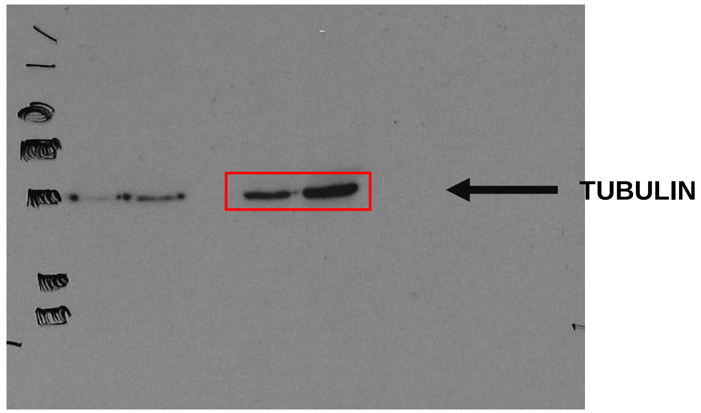

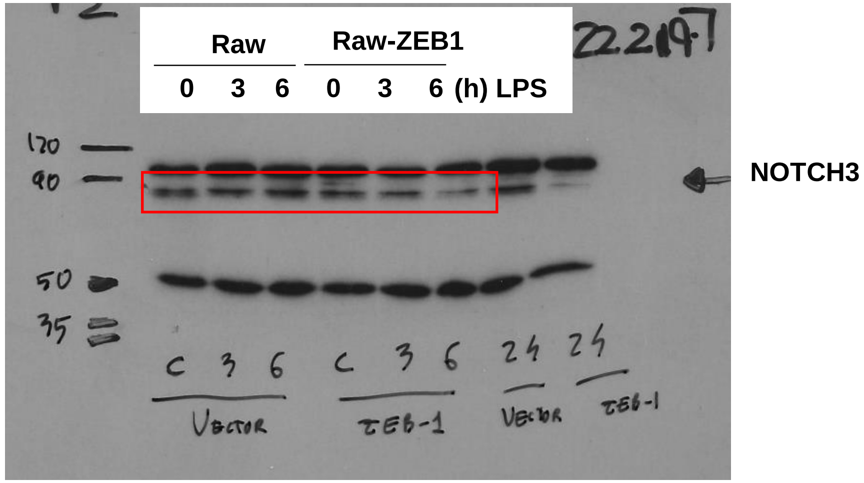

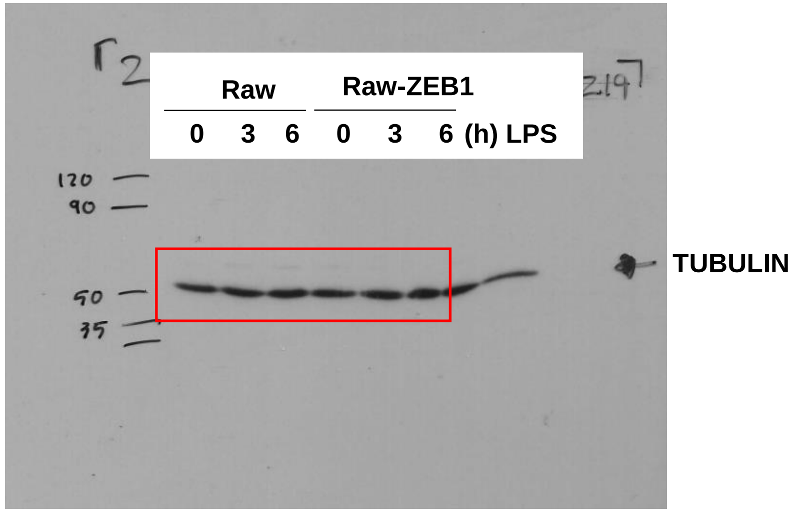

Following are the full-length immunoblots for **figure 5B**

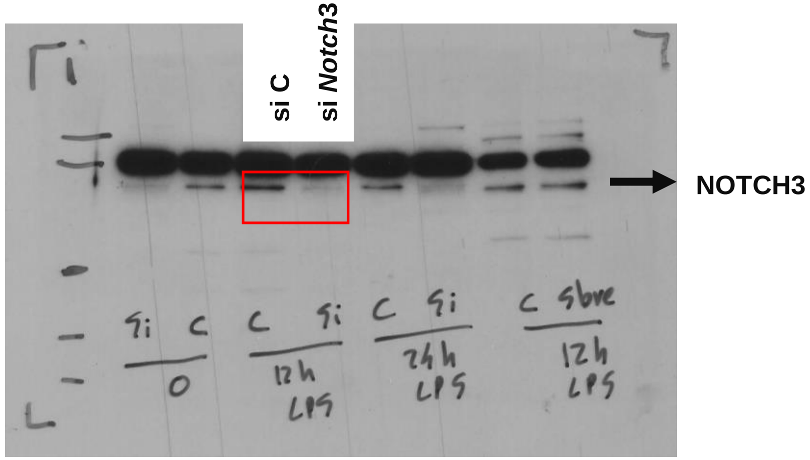

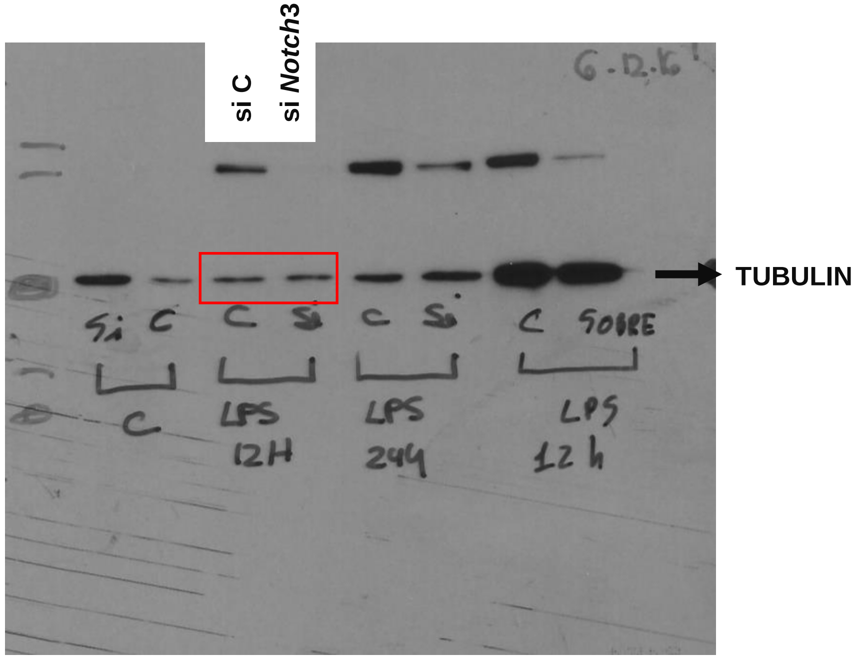

Following are the full-length immunoblots for **figure 5C**

0 12 24 0 12 24 (h) LPS

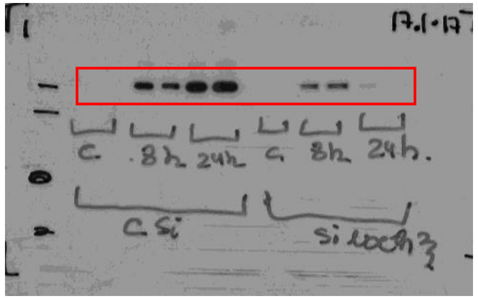

iNOS

0 12 24 0 12 24 (h) LPS

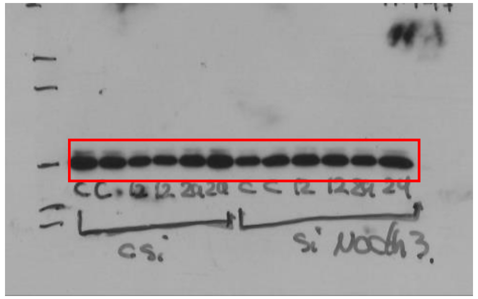

TUBULIN

Following are the full-length immunoblots for **figure 6B**

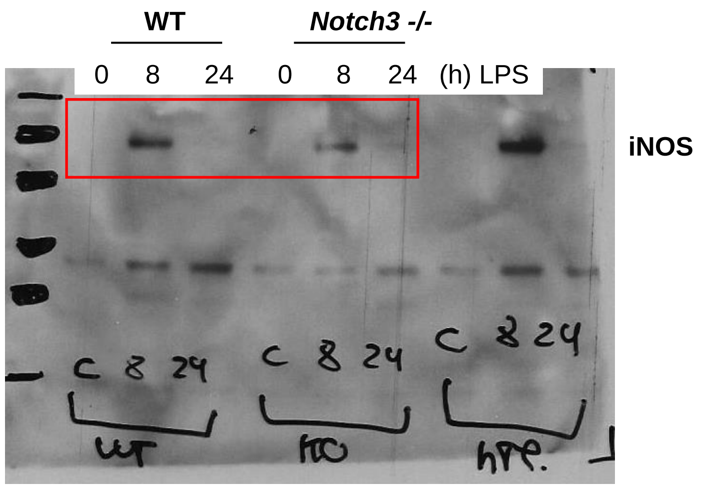

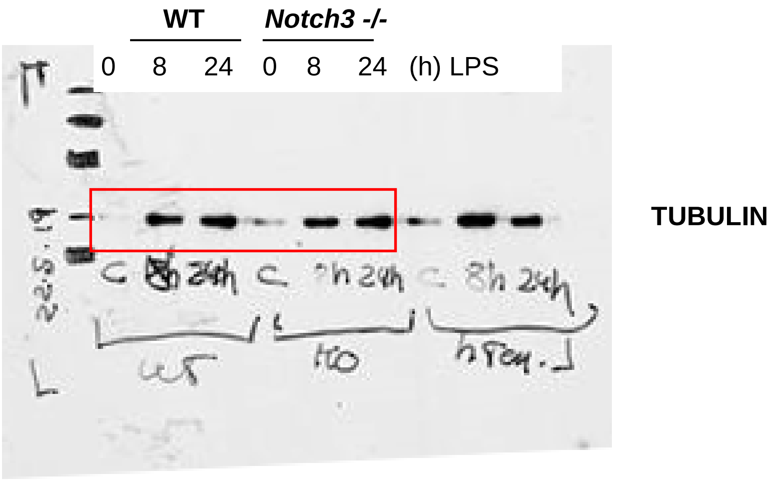

Following are the full-length immunoblots for **figure 7A**

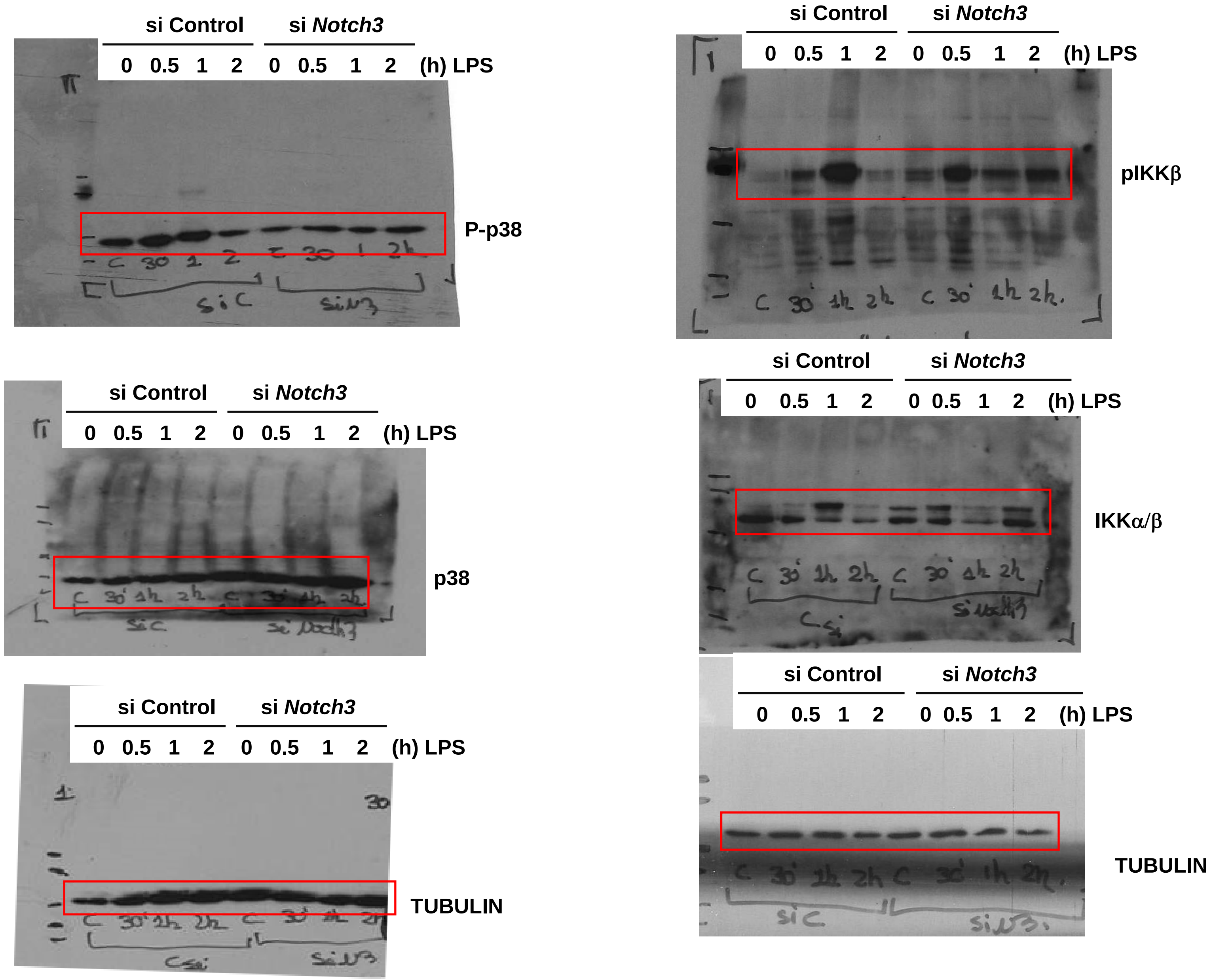

Following are the full-length immunoblots for **figure 7B**

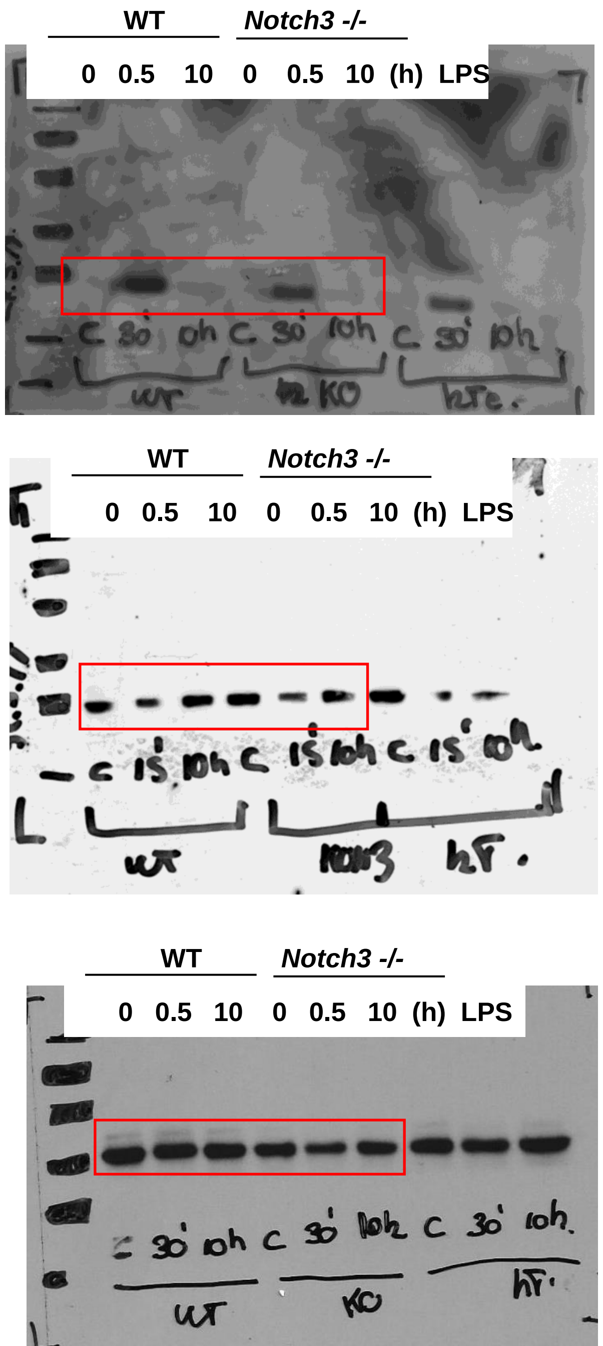

Following are the full-length immunoblots for **figure 7C**

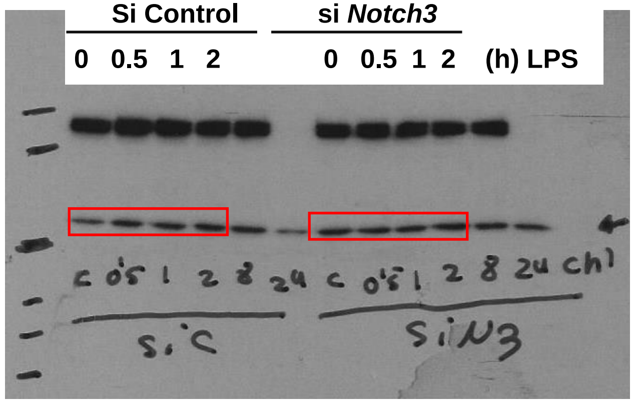

p65

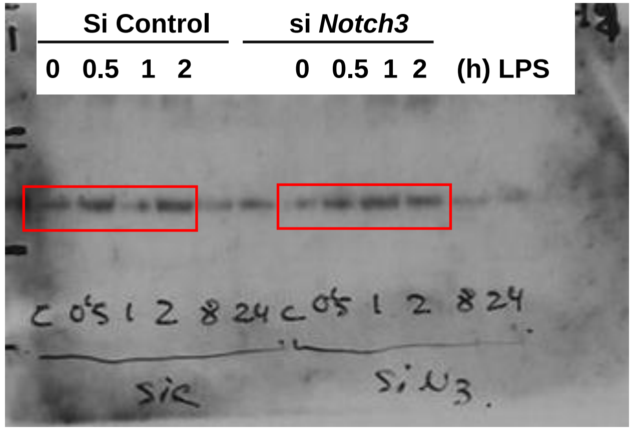

p65 Ser536

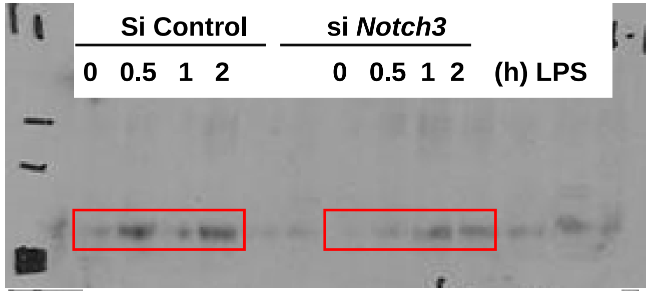

p65 Ser276

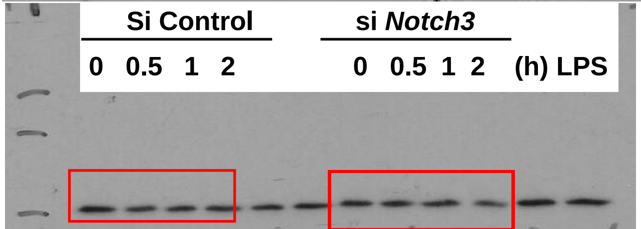

TUBULIN
